# Human IgG Subclasses Differ in the Structural Elements of Their *N*-Glycosylation

**DOI:** 10.1021/acscentsci.4c01157

**Published:** 2024-10-10

**Authors:** Weiwei Wang, Joshua C. L. Maliepaard, Timon Damelang, Gestur Vidarsson, Albert J.R. Heck, Karli R. Reiding

**Affiliations:** † Biomolecular Mass Spectrometry and Proteomics, Bijvoet Center for Biomolecular Research and Utrecht Institute for Pharmaceutical Sciences, Utrecht University, Padualaan 8, 3584 CH Utrecht, The Netherlands; ‡ Netherlands Proteomics Center, 3584 CS Utrecht, The Netherlands; § School of Pharmaceutical Science, Shanghai Jiao Tong University, 800 Dongchuan Road, 200240 Shanghai, People’s Republic of China; ∥ Sanquin Research, Immunoglobulin Research Laboratory, Amsterdam 1006 AD, The Netherlands; ⊥ Sanquin Research, Department of Experimental Immunohematology and Landsteiner Laboratory, Amsterdam 1006 AD, The Netherlands; # Sanquin Research, Department of Immunopathology, Amsterdam 1006 AD, The Netherlands

## Abstract

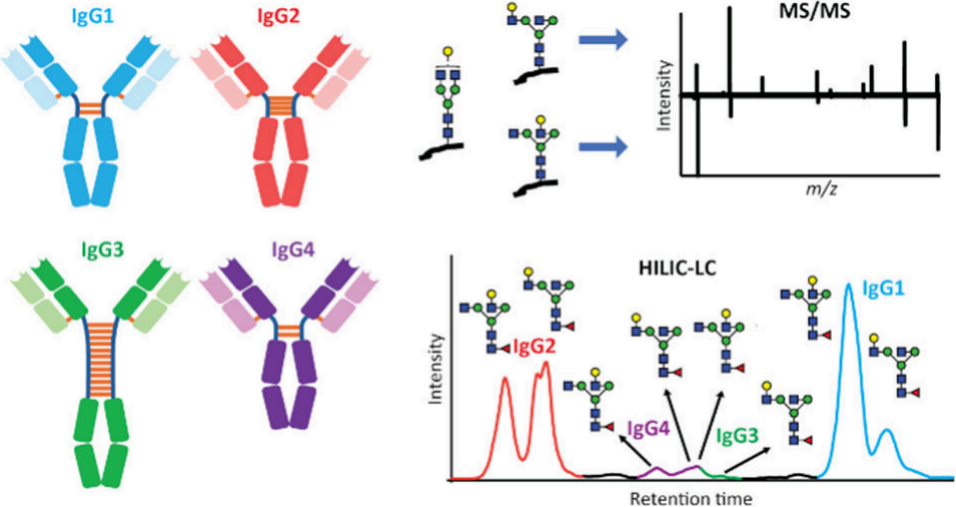

Although immunoglobulin G (IgG) harbors just one *N*-glycosylation site per heavy chain, this glycosylation plays a key role in modulating its function. In human serum, IgG is classified into four subclasses (IgG1, IgG2, IgG3, IgG4), each characterized by unique features in their sequences, disulfide bridges and glycosylation signatures. While protein glycosylation is typically studied at the compositional level, this severely underestimates the complexity of the molecules involved. Glycan functionality heavily relies on the precise linkages and branching between monosaccharides, yet these features are challenging to study. Here, by development of a nanohydrophilic interaction chromatography (HILIC)-LC-MS/MS method, we reveal distinct structural glycosylation signatures for each of the four IgG subclasses, namely that IgG1 and IgG3 display predominant galactosylation of the 6-branched antenna, IgG2 instead of the 3-branched antenna, while IgG4 displays a balance. These and other subclass-specific glycostructural elements proved observable in both recombinant and endogenous IgGs as present in human plasma, in which interindividual differences and temporal stability could be demonstrated. Structural glycoproteomics is expected to fundamentally alter the way in which we study IgG, opening up a new layer of functional investigation and biomarker development, while also revealing new key structural differences between recombinant IgG subclasses in therapeutic applications.

## Abstract 2

Structural glycoproteomics by HILIC-LC-MS/MS reveals distinctive structural glycosylation signatures across human IgG subclasses.

## Introduction

**Figure 1:**
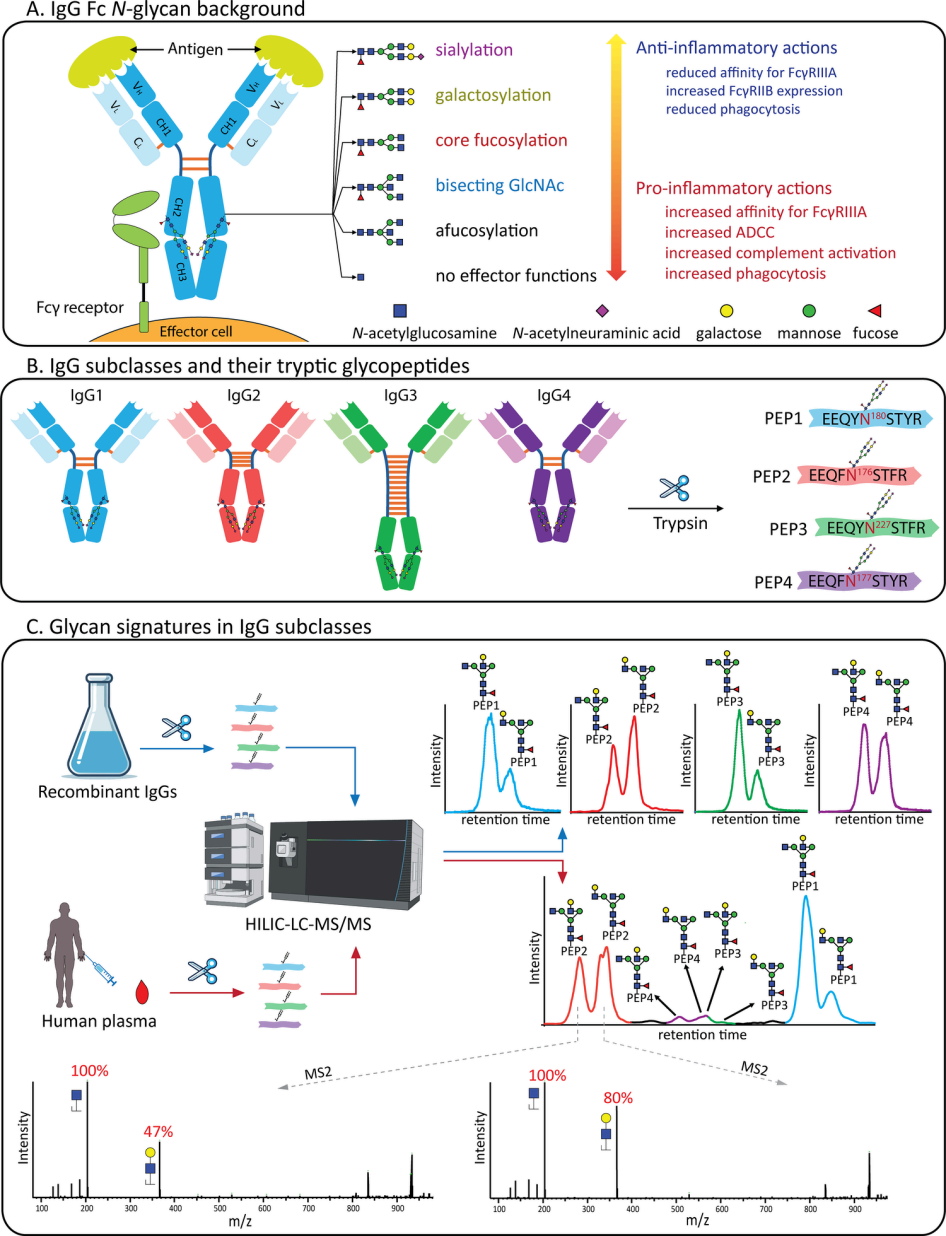
Compositional and structural traits of IgG *N*-glycosylation across IgG subclasses and its impact on IgG function. A) The glycan structures of the Fc domain in IgGs are critical for its function, as variations in these structures lead to different anti-inflammatory and pro-inflammatory actions. B) IgGs are composed of four subclasses, which differ in their peptide sequences, hinge regions, and glycan compositions. Upon trypsin digestion, these IgG subclasses generate similar glycopeptides with comparable peptide sequences. These glycopeptides can be effectively separated and identified using HILIC-LC-MS/MS, based on their glycan structures. C) We observe that the four IgG subclasses exhibit distinct glycan signatures, and these signatures are consistently observed in both rIgGs and human plasma.

Immunoglobulin Gs (IgGs) represent some of the most abundant (glyco)proteins in human serum, with an average concentration of 10 mg/mL,^1^ having critical functions in the humoral immune response. The IgG antibody family consists of four subclasses, namely IgG1, IgG2, IgG3, and IgG4 (Figure 1). This classification in partly based on differences in the protein sequences observed within the constant region of the heavy chain, but the four subclasses distinctly differ in more structural and functional aspects.^2^ Among other things, they differ in their effector functions via interactions with IgG-Fc receptors (FcγRs) and complement C1q, with IgG1/3 typically having the highest affinities and IgG2/4 the lowest.^3^ Besides variations in the sequence, IgG subclasses differ in structural elements such as the amount and position of inter- and intrachain disulfide bridges, as well as the length and flexibilities of the hinge-regions.

Notably, all IgG subclasses harbor a conserved *N*-glycosylation site at Asn297 in the CH2 domain. Although these sites are highly conserved, the glycosylation they harbor can be distinct and was shown to affect IgG functionality.^4^ For IgG1, it is known that glycan specificities modulate antibody-dependent (AD) functions, including cellular cytotoxicity (ADCC),^5^ phagocytosis by cells (ADCP),^6,7^ as well as complement deposition (ADCD).^8^ For instance, absence of core-fucosylation enhances ADCC,^9^ while increased galactosylation boosts ADCP^10^ and increased sialylation reduces ADCD.^11^ Moreover, in the context of therapeutic antibodies, the glycosylation of the Fc-domain can significantly impact IgG pharmacokinetics, including half-life, stability, solubility, safety, and tolerability of the drug.^12^ Thus, a comprehensive understanding and control of IgG glycosylation is key for the design of antibody drugs.^13^

For analysis of protein *N*-glycosylation, it is critical to distinguish a glycan’s compositional characteristics (monosaccharide count) from the structural features (monosaccharide sequence, glycosidic linkages, etc.).^14^ While hard to study, also the structural elements of glycosylation, just like the composition, will have large consequences for glycoprotein function. For instance, sialic acid linkage serves as a species barrier for virus infection, e.g.., bird influenza binding upper tracheal α2,3-linked sialylation while humans display α2,6-linked sialylation instead,^15^ while the effectivity of IVIG treatment requires α2,6-linked sialylation and not the α2,3-linked alternative.^16^ Even though our knowledge about the composition of IgG Fc glycans is well-developed, a notable gap in structural understanding persists. This is especially the case for the different subclasses of IgG, which need to be analyzed at the glycopeptide level to be reliably differentiated.

Various mass spectrometric (MS) methodologies have been developed for the characterization of IgG glycosylation, including liquid chromatography–mass spectrometry (LC-MS),^17^ capillary electrophoresis-mass spectrometry (CE-MS)^18^ and matrix-assisted laser desorption/ionization time-of-flight mass spectrometry (MALDI-TOF-MS).^19,20^ There are two main principal strategies in MS-based approaches, each with its own advantages and disadvantages: (1) purifying all IgG for glycomics analysis can yield glycostructural information, but does not enable the association of these structures with specific subclasses, the determination of their origin in terms of Fab- or Fc-regions, or even their distinction from impurities;^21,22^ (2) isolating individual IgG subclasses and examining glycopeptides or Fc/2 subunits provides insights into the glycosylation, but only at the compositional and not the structural level.^20,23,24^ It is apparent that a comprehensive method to study glycan structure at the glycopeptide level remains urgently needed.

As such, by combining recent innovations in LC^25,26^ and MS,^27^*i.e*., high-resolution nano-HILIC-LC and structure-dependent MS/MS, we here present analytical methodology capable of quantifying IgG glycopeptides, within complex samples, at the glycostructural level. Using this methodology, we demonstrate that IgG subclasses carry differential yet defined structural glycan characteristics, in both recombinant and endogenous plasma IgGs. Furthermore, these structural characteristics appear unaffected by allotypic variation, while at the same time being longitudinally stable in healthy individuals. Following these investigations, we postulate that IgG glycan structures are conserved and unique within the different subclasses, our technology now enabling the study of its functional consequences and biomarker capabilities.

## Results and Discussion

Although the CH2 domain *N*-glycosylation is highly conserved between IgG subclasses, we hypothesized that there would be distinct subclass-specific differences in both the compositional and structural buildup of the attached glycans, that these would differ between individual serum donors, and that there would be a discrepancy between endogenous serum and recombinant IgGs. To test these hypotheses, we set out to develop an analytical method that could both qualitatively and quantitatively assess compositional and structural *N*-glycosylation features of each IgG subclass and tackle serum samples extracted from single or pooled donors.

Our approach combined two key analytical features, namely (1) high-resolution nano HILIC-LC for the efficient separation of glycopeptide compositional and structural isomers, and (2) utilizing higher-energy collisional dissociation (HCD)-based MS/MS fragmentation for the precise identification of the glycan composition and topology on the observed glycopeptide isomers.^27^

### Method Performance on Recombinant IgG1

**Figure 2:**
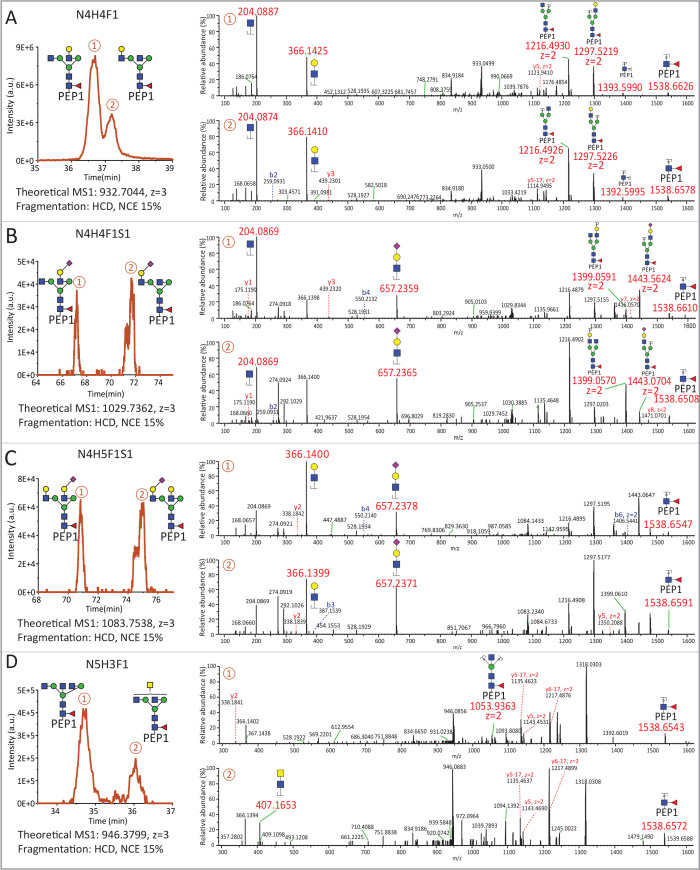
HILIC-based separation and tandem mass spectrometric assignment of four pairs of tryptic *N*-glycopeptide isomers from rIgG1 produced in human HEK293F cells. From top to bottom, data are shown for the same glycopeptide (sequence: EEQYNSTYR) harboring *N*-glycan compositions A) N4H4F1, B) N4H4F1S1, C) N4H5F1S1, and D) N5H3F1 (N = *N*-acetylhexosamine, H = hexose, F = fucose, S = *N*-acetylneuraminic acid). The structural traits were assigned based on the intensity-based characteristics of oxonium ions observed in the corresponding MS/MS spectra.

Recombinant IgG1 (rIgG1) was purified from HEK293F cells and digested with trypsin prior to analysis by nano-HILIC-LC-MS/MS, resulting in the formation of glycopeptides sharing the EEQYNSTYR backbone (termed PEP1, where 1 indicates IgG1). The MS/MS data were searched using Byonic (detailed glycopeptide backbone fragmentation is shown in Figure S1), with manual verification of the search results. Notably, using HILIC separation we observed multiple PEP1 glycopeptides that were isobaric, *i.e*., exhibiting the same apparent mass (see Figure 2). With chromatographically separated glycopeptide isomers, we argued that MS/MS fragmentation profiles would disclose unique ratios in abundances of oxonium ions, indicative of specific structures in their branching, as shown by our lab before.^27^

In Figure 2A, two fragmentation spectra are depicted from chromatographically separated PEP1 glycopeptide isomers harboring a N4H4F1 glycan (N = *N*-acetylhexosamine, H = hexose, F = fucose, S = *N*-acetylneuraminic acid), whereby the MS/MS spectra revealed their unique features. The EEQYNSTYR rIgG1 glycopeptide carrying the monogalactosylated *N*-glycan (N4H4F1) clearly separated into two peaks with different intensities by HILIC chromatography (Figure 2A). By subjecting these two peaks to HCD fragmentation (normalized collision energy, NCE, of 15%), we could detect divergent fragmentation patterns. While the first peak displayed a ratio between N1H1 (*m*/*z* 366.1425) and N1 (*m*/*z* 204.0887) ions of 0.47 to 1.00, the second peak displayed a ratio of 0.80 to 1.00 instead. Having previously reported a systematic preference for 3-branch fragmentation (the branch starting at the α1,3-linked mannose) at low collision energies,^27^ we assigned the first PEP1 peak as carrying the galactose at the 6-branch (α1,6-linked mannose) and the second PEP1 peak to having the galactose at the 3-branch. Notably, the ratio difference between these two isomers was in good agreement with what has previously been reported for rIgG1, based on analysis of the released glycans.^28^

The HILIC chromatography also allowed us to baseline separate several other PEP1 glycopeptides carrying monosialylation (both N4H4F1S1 and N4H5F1S1, Figure 2B, 2C)). In the MS/MS spectra of these distinct glycopeptides, the first chromatographic peak consistently showed lower relative N1H1S1 (*m*/*z* 657.2359) to either N1 (0.28:1.00) or N1H1 (0.32:1.00) when compared to the second chromatographic peak (0.79:1.00 and 0.86:1.00). Thus, based on our prior knowledge,^27^ we assigned both first peaks as having the sialylation at the 6-branch, and both second peaks as having the sialylation at the 3-branch.

In addition, HILIC chromatography allowed us to separate HexNAc positions, as apparent from the PEP1 glycopeptide with a N5H3F1 composition (Figure 2D). The MS/MS spectra of these peaks revealed clear ions for either a bisecting GlcNAc (peptide with N3H1F1, *m*/*z* 1053.9363) or an antennary LacdiNAc structure (N2 fragment, *m*/*z* 407.1653). Potential branch isomerism of the LacdiNAc structure proved to be outside the limit of detection of our measurement. While not likely to be found in endogenous human IgG1, LacdiNAc is a well-known feature of HEK293F-produced recombinant IgG proteins.^29^

### IgG Subclasses Expressed in HEK293F Cells Display Distinct Structural Glycan Signatures

**Figure 3:**
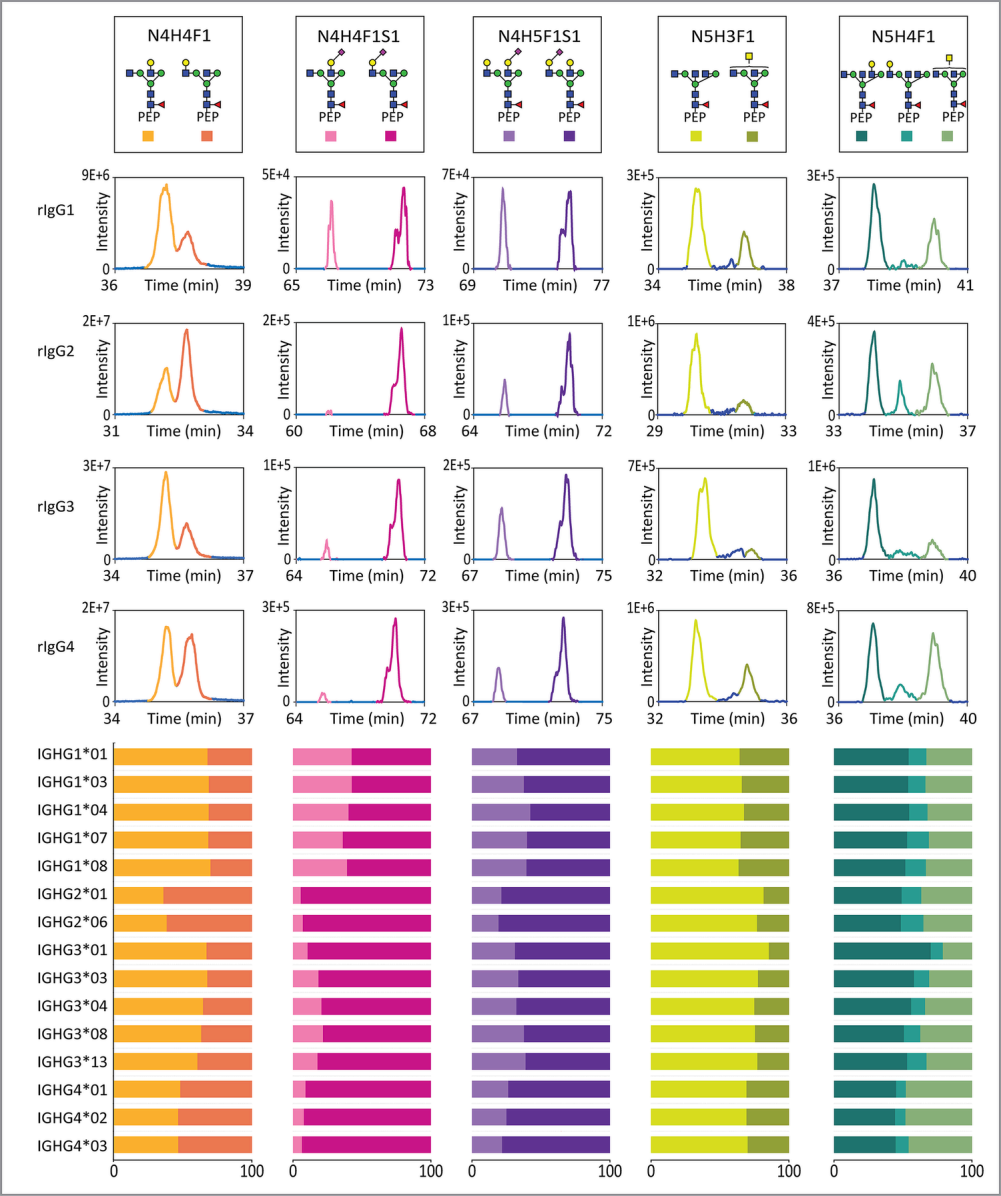
Comparison of recombinant IgG subclasses and allotypes. HILIC-based chromatographic separation of five pairs of tryptic *N*-glycopeptide isomers, as depicted at the top, with compositions N4H4F1, N4H4F1S1, N4H5F1S1, N5H3F1, and N5H4F1. The relative abundances of the structural isomers were extracted from the nano-HILIC-LC-MS/MS data and summarized as bar graphs at the bottom of the figure. The data was obtained from 5 allotypes of IgG1, 2 allotypes of IgG2, 5 allotypes of IgG3, and 3 allotypes of IgG4, all produced recombinantly in HEK293F cells.

Being able to separate and assign and quantify structural isomers of IgG1 glycopeptides, we next set out to test whether other IgGs revealed alike or distinct glycan traits. To investigate the glycosylation differences across IgG subclasses and allotypes, we analyzed tryptic glycopeptides from recombinant IgG subclasses and allotypes (with listed differences in amino acids,^5^ shown in Table S1) derived from HEK293F cells. As *N*-glycosylation on the Fc domain of IgGs subclasses is highly conserved, the amino acid sequences of the tryptic glycopeptides are alike but can nevertheless be distinguished (EEQYNSTYR for IgG1, EEQFNSTFR for IgG2, EEQYNSTFR for IgG3, and EEQFNSTYR for IgG4, the amino acid differences underlined). As with the rIgG1 sample described above, also for the other rIgG subclasses we observed chromatographic separation of glycopeptide isomers (Figure 3). Quantifying the resulting peak areas using Skyline (Figure 3, Figure S2), we achieved the relative quantification of more than a dozen glycopeptide structures for each rIgG subclass (Figure S2).

Although the glycan quantification of the rIgG subclasses was similar at a compositional level, comparison of the structures underlying these compositions revealed subclass-specific signatures. For instance, glycopeptides with the N4H4F1 motif in rIgG1 and rIgG3 demonstrated a distinct preference of 6-branch galactosylation (70% for rIgG1, 67% for rIgG3) over 3-branch galactosylation (30% for rIgG1, 33% for rIgG3), with. In contrast, rIgG2 exhibited a higher relative prevalence of 3-branch galactosylation (64%), while rIgG4 showed approximately equal abundances of both (47% for 6-branch galactosylation, 53% for 3-branch galactosylation).

Interestingly, while sialylated glycan compositions such as N4H4F1S1 and N4H5F1S1 typically featured a single sialic acid predominantly positioned on the 3-branch, subclass-specific differences in the abundance ratios were evident (see the second and third columns of Figure 3). For example, for N4H4F1S1, rIgG1 exhibited a significantly higher occurrence of 6-branch sialic acid (42%), dramatically surpassing the other subclasses (with no more than 2%). In contrast, for N4H5F1S1, both rIgG1 and rIgG3 showed a greater proportion of structures with 6-branch sialylation (42% for rIgG1, 38% for rIgG3), in comparison to rIgG2 and rIgG4, which have no more than 20%. For rIgG glycopeptides containing N5H3F1 or N5H4F1, we observed both bisection and LacDiNAc structures across all subclasses, with the bisected structures showing a notably higher relative abundance. For the N5H3F1 variant, rIgG2 and rIgG3 demonstrated a higher proportion of bisection (around 80%), as compared to rIgG1 and rIgG4 which displayed no more than 70%. rIgG4 presented the lowest prevalence of bisection (approximately 50%), which was substantially lower than the other subclasses (no less than 65%). Intriguingly, for glycopeptides featuring the N5H4F1 glycan, there was a consistently higher 3-branch galactosylation for the bisected structures, compared to the nonbisected structures, across all subclasses. Based on these findings we concluded that different IgG subclasses, when produced recombinantly in HEK293F cells, exhibited unique ratios of *N*-glycan structures.

Next to subclasses, differences exist between IgGs in the form of allotypes: genetic variants in the constant regions that are often different between individuals and populations. To ascertain the glyco-structural effects of several of these allotypes, we analyzed 5 recombinantly produced variants of IgG1, 2 of IgG2, 4 of IgG3, and 3 of IgG4 (Table S1).^5^ From these 14 different rIgG we observed that all allotypes exhibited nearly identical structural characteristics within a subclass, but that these consistently differed between subclasses (Figure 3 and Figure S2).

### Human Plasma IgG Subclasses Exhibit Distinct Structural Glycan Signatures

Although HEK293F cells are of human origin (*i.e*., human cells) it is well-known that *N*-glycosylation profiles of recombinantly produced proteins may be quite different from those that are present *in vivo* in the blood.^30^ Because of this, next we explored whether our nano-HILIC-LC-MS/MS method could be applied to analyze the *N*-glycan signatures of IgG subclasses as present in human plasma. To achieve this, we directly analyzed IgG glycopeptides from a tryptic digest of plasma, which is evidently more challenging by having the whole plasma proteome as background. Fortuitously, IgGs are quite abundant in plasma, an advantage for the detection of their *N*-glycopeptides, although this is more the case for IgG1 and IgG2, and less so for IgG3 and IgG4.

Next to the background, the study encountered the issue that several of the glycopeptides shared masses. A notable example of this was IgG3 PEP3 (EEQYNSTFR) with N4H3F1, IgG4 PEP4 (EEQFNSTYR) with N4H3F1 and IgG2 PEP2 (EEQFNSTFR) with N4H4, all sharing a mass of 2617.0437 Da. Fortunately, this additional form of glycopeptide isomerism could also be separated using the optimized nano-HILIC-LC-MS/MS approach, meaning that all IgG glycopeptides could be successfully identified through MS and MS/MS (Figure S3). The high-resolving nano-HILIC-LC-MS/MS method therefore enabled the simultaneous direct analysis of the Fc-domain *N*-glycosylation profiles of the co-occurring IgG subclasses in human plasma, eliminating the necessity for any further purification or enrichment.

**Figure 4:**
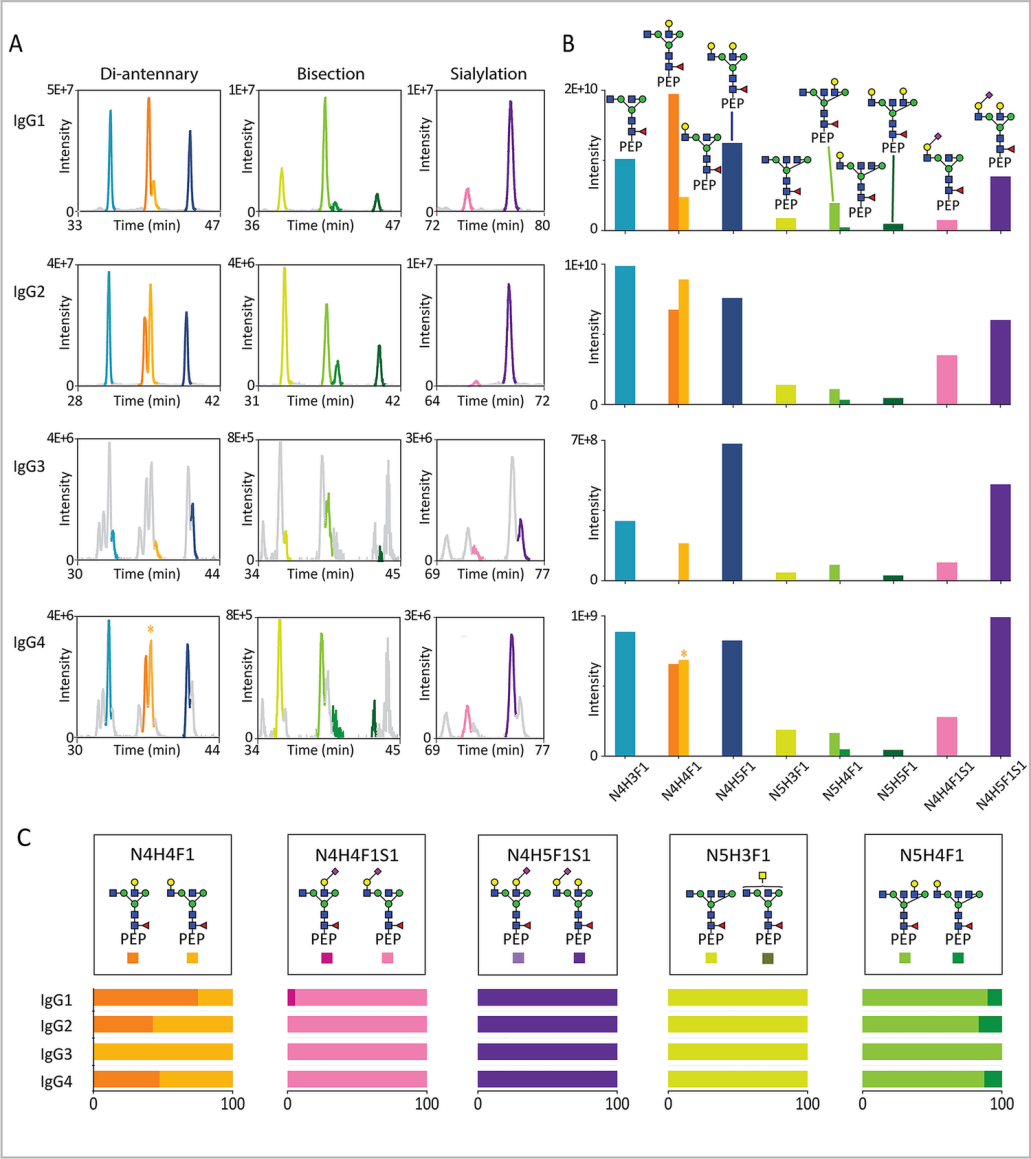
Structural *N*-glycosylation as observed in IgG subclasses obtained from a pooled plasma sample of 20 donors. A) HILIC-based chromatographic separation of *N*-glycopeptide isomers from IgG subclasses as extracted from the pooled plasma sample. B) Predominant compositional and structural traits observed in IgG subclasses in pooled human plasma. C) Relative abundance of distinct structural traits within isomeric IgG *N*-glycopeptides. Here, the chromatographic peaks of glycopeptide (EEQYNSTFR) with N4H4F1 (6-branch galactose) from IgG3 and glycopeptide (EEQFNSTYR) with N4H4F1 (3-branch galactose) overlapped. We attributed the entire area of the overlapping peak to IgG4, due to the higher abundance of IgG4 compared to IgG3. We have marked this overlapping as an asterisk (*) in our analysis.

To start, we set out to analyze the IgG glycopeptides from a pooled plasma sample originating from 20 healthy donors (Figure 4 and Figure S4). As expected, the general plasma *N*-glycosylation profiles were already rather distinct from those observed for the corresponding recombinant IgGs (as produced in HEK293F cells), with notably diminished glycan diversity in plasma, as well as an absence of LacdiNAc motifs. Aligning with literature on subclass abundance,^31^ we observed very intense IgG1 and IgG2 signals, and lower glycopeptide abundances for IgG3 and IgG4. Examining compositional glycan profiles for each subclass already revealed some subtle differences, for instance an increased sialyation of IgG2 (21%), IgG3 (30%) and IgG4 (26%), compared to IgG1 (14%) (Figure S5).

Focusing on the relative abundance of structural isomers, as uniquely elucidated by our nano-HILIC-LC-MS/MS method, we observed that human plasma IgG subclasses indeed displayed unique structural abundance patterns (Figure 4). Remarkably, these patterns matched those observed in rIgGs, albeit with some notable exceptions. Akin to their recombinant counterparts, plasma IgG1 was dominated by 6-branch galactosylation (Figure 4AB), while IgG2 preferentially displayed 3-branch galactosylation instead (Figure 4AB). For IgG1 N4H4F1 specifically, released glycan analysis previously found matching branch ratios, strengthening the reproducibility of the findings.^32^ However, our peptide-centric approach can now rule out interference by the other subclasses, and indeed, characterize these others as well. While IgG3 and IgG4 did show overlapping peaks, applying the pattern ratios of rIgG3 and rIgG4 N4H4F1 to plasma suggested highly dominant 6-branch galactosylation in IgG3, whereas IgG4 displayed a more equal abundance of both branches. For bisected glycans, a 6-branch galactose structure predominated across all IgG subclasses, with the 3-branch structure being less abundant or not detected (IgG3), both in human plasma and rIgGs.

Surprisingly, and in contrast to what we observed for recombinant IgGs, the sialylated glycopeptides in plasma IgGs displayed a single structure across all subclasses, favoring either the 3-branch sialic acid for nonbisected glycans (Figure 4) or the 6-branch for bisected glycans (Figure S5). To recall, recombinant IgGs displayed high abundances of both 6-branch and 3-branch sialylation in their monosialylated glycans. MS1 signals potentially belonging to disialylated glycopeptides were detected in the human plasma as well, but their low signal intensity precluded the necessary MS2 quality to unambiguously make the determination.

### Glycan Structures on Human IgG Display Individual Uniqueness with Temporal Stability

**Figure 5:**
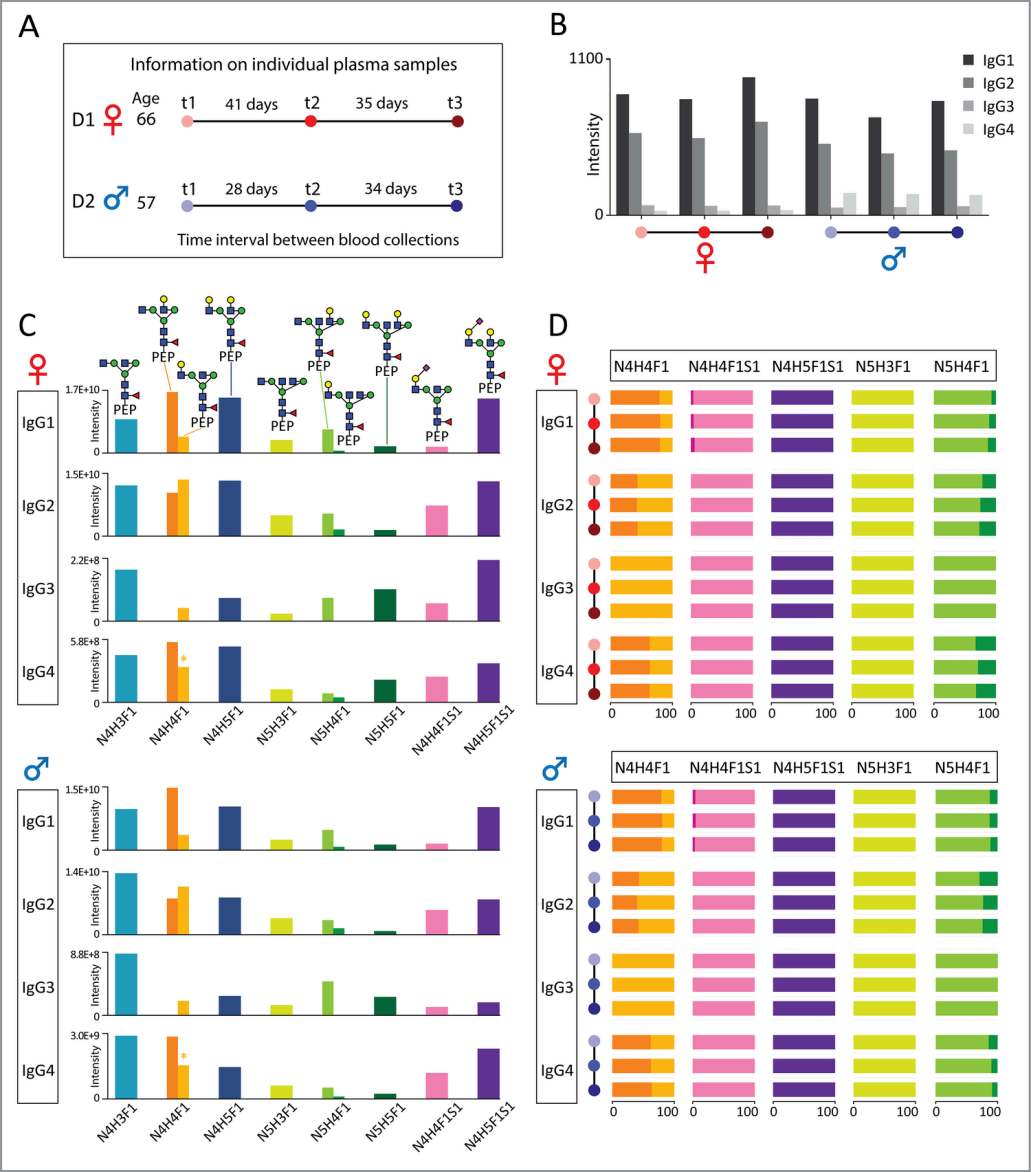
Subclass-specific IgG glycosylation obtained from two individual donors at 3 distinct time points of sampling. A) Donor and sampling characteristics. B) Relative abundance of the four IgG subclasses within the plasma of donor D1 and D2 and the three sampled time points. Quantitation is based on LFQ proteomics data using only unique peptides of the IgG subclass Fc regions. C) Predominant compositional and structural traits observed in IgG subclasses in donor D1 and D2. D) Relative abundance of distinct structural traits within isobaric IgG *N*-glycopeptides as observed for donor D1 and D2, and the three sampled time points. The asterisk (*) indicates overlapping peak areas of IgG3 and IgG4, attributed to IgG4 due to its generally higher abundance.

Plasma protein glycopeptides have long been proposed as diagnostic biomarkers for diseases, but this does require a stable background to compare against.^33,34^ We therefore next explored to which degree IgG structural *N*-glycosylation would vary among individuals and in time. To achieve this, we obtained three plasma samples from two healthy donors (D1, female, age 66 and D2, male, age 57), sampled at approximate intervals of a month (Figure 5A). These were subsequently investigated using our nano-HILIC-LC-MS/MS methodology.

As already expected from the pooled plasma sample and the literature,^35^ IgG1 (52%–56%) and 2 (31%–38%) were most abundant in the donor samples, with IgG3 (4%) and 4 (2%–10%) being of lower abundance as determined by proteomics (Figure 5B). Furthermore, the proteomics results of the IgG subclasses were consistent with the subclass-specific glycopeptide peak area results (Figure S6). Notably, D2 had a relative higher abundance of IgG4 (10%), when compared to D1 (2%). The temporal abundances of the IgG subclasses mostly appeared constant within a given donor.

From comparing the samples by structural glycoproteomics we extracted the following information: 1) overall, the compositional and structural *N*-glycosylation features in individual plasma samples closely matched those observed in the pooled human plasma sample (Figures 4 and 5, respectively). 2) Next to unique subclass features, we observed differences between D1 and D2, *e.g*., D2 showing a higher proportion of N4H3F1 (16% in IgG1 and 23% in IgG2) compared to D1 (12% in IgG1 and 15% in IgG2) (Figure 5C). 3) The relative ratio of glycan isomers within the same composition showed subclass-specific signatures consistent with those observed in the pooled plasma (Figure 5D). 4) The relative abundance of the compositional and structural features across all subclasses remained largely identical over time. While we only studied samples covering a 3-month period and glycan remodelling could still occur across larger timeframes, it was remarkable to see such stability. It must be noted that the plasma lifetime of individual IgG molecules is much shorter than 3 months.^36^ Specifically, the half-life for IgG1, IgG2, and IgG4 is around 21 days, while for IgG3, it is approximately 7 days.^1^ Therefore, the structural glycosylation profile must be stably inherited from one generation of IgGs to the next. This points at strong glycosylation control within IgG-producing cells (plasma- and B-cells), or efficient remodelling, recycling and/or degradation of IgG glycoforms already in the circulation.

## Conclusions

We here developed and employed nano-HILIC-LC-MS/MS methodology to separate and characterize compositional and structural variants of tryptic Fc glycopeptides derived from IgG subclasses. This approach enabled a comprehensive qualitative and quantitative analysis of IgG *N*-glycosylation prevalent across the distinct human IgG subclasses, delineating unique distinctive signatures among the four IgG subclasses that are only visible when the structural elements of glycosylation are considered. In summary, our data, covering both rIgGs from HEK293F cells and endogenous IgGs derived from human plasma, exposed several novel aspects of the glycan profiles of IgGs: 1) each IgG subclass possesses a distinctive structural glycosylation signature, 2) individual donors possess unique quantitative differences in their IgG glycosylation that are stable in time (at least for the 3-months window here covered), and 3) the glycosylation profiles are quite divergent between recombinant and human plasma IgG.

Our research and methodology is poised to significantly advance several key areas, for example, the insight into the relationships between IgG glycoproteoforms and their functional roles in mediating immune responses.^37^ For instance, IgG1 and IgG3 are recognized as robust activators of Fc-mediated effector mechanisms, such as ADCC, CDC, and ADCP, while IgG2 and IgG4 tend to elicit more subtle immune responses. Notably, both IgG1 and IgG3, as well as IgG2 and IgG4, display similar quantitative traits in their glycan structures, including the galactosylation of the 3-branch and 6-branch, (e.g., of N4H4F1). This pattern prompts further exploration of the potential links between specific glycan structures and their corresponding functional attributes. On a fundamental level, it will need to be assessed what drives the differences in antibody glycan structure, even for IgG subclasses that are produced in the same cell system. The answer to this could range from a dependency on adjacent peptide sequences, structure-dependent chaperoning, or indeed specific remodelling, recycling, or breakdown of IgGs depending on the glyco-structural elements.

The advent of structural glycoproteomics will enhance our comprehension of glycan diversity in different biological sources, thereby assisting in the development and optimization of antibody therapeutics and vaccine formulations. At the same time, the temporal stability of the IgG glycosylation, witnessed within the presumably healthy controls, lends significant opportunity for investigating diseases in which a deviation from homeostasis is to be expected. While a diseased or immortalized cell may perform galactosylation and sialylation like a healthy one, it is unlikely to do so in equivalent structural ratios.

## Materials and Methods

### Chemicals and Materials

Chloroacetamide (CAA), Tris(2-carboxyethyl)phosphine hydrochloride (TCEP), Tris, trypsin, Lys-C, sodium deoxycholate (SDC) and sodium acetate were obtained from Sigma–Aldrich (Steinheim, DE). Milli-Q water was generated from a Merck Milli-Q IQ 7003 system (Darmstadt, DE). The Oasis PRiME HLB plate was purchased from Waters (Etten-Leur, NL). Acetonitrile and 0.1% formic acid (FA) were obtained from Biosolve (Valkenswaard, NL). Trifluoroacetic acid (TFA) was obtained from Honeywell International Inc. (Charlotte, NC). Pooled human plasma sample from 20 donors was obtained from Affinity Biologicals (Ancaster, Canada) with a product number of UFNCP0125. Longitudinal EDTA plasma samples from two healthy Caucasian donors (D1 and D2) with the identifier of 7005–8200 were obtained from Precision Med (Solana Beach, CA, US).

### Human Plasma and Protein Digestion

Human plasma samples or rIgGs protein (expressed by transient transfection of heavy anti-RhD/anti-TNP) and light chain (anti-RhD/anti-TNP) in HEK293F suspension cells)^5^ were diluted in digestion buffer containing 100 mM Tris–HCl (pH 8.5), 1% *w/v* sodium deoxycholate (SDC), 5 mM TCEP and 30 mM CAA. Lys-C was then added to digest plasma or proteins for 3 h at an enzyme-to-protein ratio of 1:75 (*w/w*) at 37 °C, and the resulting peptide mixtures were further digested overnight at 37 °C by trypsin (1:20; *w/w*). The next day, SDC was removed via acid precipitation (0.5% trifluoroacetic acid) (TFA). The peptides were desalted using an Oasis PRiME HLB plate then dried and stored at −80 °C before MS analysis.

### HILIC Column Preparation

The preparation of the HILIC column involved two steps: 1) the production of the frit-capillary, and 2) HILIC column packing. For step 1): the frit solution was prepared in a glass vial by mixing 300 μL potassium silicate (PQ Europe) with 100 μL formamide (Merck). After adding formamide, the solution was vortexed immediately. After that, one end of the capillary (75 μm ID, 40 cm) was dipped in the frit solution for a few seconds to provide a frit of ∼1 cm. To check whether the frit was transported into the capillary, we used a microscope (Olympus) with a separate source of light (Schott, KL 1500). The capillaries were placed in a clean glass vessel with the frit end facing down and put in an oven for 1 h at 100 °C. During this step, the frit was polymerized. For step 2): capillaries with a frit on one end were washed twice with methanol. A slurry was then prepared by adding HILIC material (HALO penta-HILIC, 2.7 μm diameter, Advanced Materials Technology, USA). to ACN with 0.1% FA, which was subsequently packed into the capillary under a helium pressure of 100 bar with magnetic stirring. The capillary column was then consolidated at a liquid pressure of 800 bar (water or 80% methanol), trimmed to 25 cm, and set aside for use.

### LC-MS Method

Shotgun LC–MS/MS was performed by means of an UltiMate 3000 UHPLC system (Thermo Fisher Scientific) coupled to an Orbitrap Fusion or an Orbitrap Exploris 480 mass spectrometer (Thermo Fisher Scientific). A total of 600 ng of the protein digests or 0.1 μL of plasma digests were loaded on a trap column (PepMap Neo Trap Cartridge, 174500, 300 μm inner diameter, 5 mm) (Thermo Fisher Scientific) coupled to a 75 μm inner diameter 25 cm analytical column (in-house packed with HALO penta-HILIC, 2.7 μm diameter) (Advanced Materials Technology, USA). The mobile-phase solvent A consisted of 0.1% FA in water, and the mobile-phase solvent B consisted of 0.1% FA in ACN.

Trapping was performed at a flow rate of 30 μL/min for 1 min with 0% B and peptides were eluted using a flow of 300 nL/min for 130 min with 95–78% B over 7 min, 78–60% B over 77 min, 60–20% B over 25 min, 20% B for 5 min and finally held at 95% B for 15 min.

For MS settings, the source voltage was set at 2000 V, and the ion transfer tube temperature was maintained at 275 °C.The initial scan parameters were established with a scan range of *m*/*z* 350–2000 and a resolution of 120,000, utilizing automatic gain control (AGC) set to standard with the maximum injection time of 50 ms. Ions with an intensity over 5 × 10^3^, with charge states from 2+ - 8+ were selected for HCD fragmentation with a dynamic exclusion time of 6 s. For HCD fragmentation, MS/MS acquisition was conducted in the HCD cell, with readings captured in the Orbitrap mass analyzer. This setup provided a resolution of 60,000 for *m*/*z* ranging from 120 to 4000, AGC set to standard, while maintaining a maximum injection time of 50 ms and a normalized collision energy of 15%. Additionally, a target mass list was utilized, encompassing various IgG glycopeptides as detailed in Supplementary Data Excel file 2.

### Data Analysis

The raw files obtained from the LC-MS/MS experiments were processed using Byonic (version 4.3.4, Protein Metrics Inc.) to search against the UniProt FASTA database of human IgGs (Supplementary Data file 1). The search parameters were set as follows: 1) a specific tryptic digest with a maximum of three miscleavages; 2) a fixed modification of cysteine residues (+57.021 Da) and variable modifications including methionine oxidation (+15.995 Da), phosphorylation (+79.9663 Da), conversion of glutamine to pyroglutamate (−17.0265 Da), and conversion of glutamic acid to pyroglutamate (−18.0106 Da); 3) variable glycosylation, as detailed in Supplementary Data Excel File 3, which lists the glycans used for the database search; 4) an error tolerance of 10 ppm in MS and 20 ppm in MS/MS.For the glycopeptides obtained via Byonic, the glycopeptides carrying certain glycan structures and their retention times were manually validated using Freestyle.

Skyline (v3.7.0.11317) was used to perform a relative quantification. The integrations acquired in this manner were reviewed to meet the following standards: (1) an error of ≤5 ppm to the theoretical mass; (2) exhibiting an idotp value ≥0.80 when compared with the theoretical isotopic pattern; (3) elution time falling within a range of ±2 min around the average retention time for the corresponding peptide; and (4) no observable instances of overlapping isotopic patterns. Here, only glycopeptides accurately identified in Freestyle were permitted for quantification using Skyline. The determination of chromatographic peaks needed to strictly adhere to retention time regulations. For baseline-separated individual peaks with a width of 30 s, the peak area was determined within this time frame. For peaks that did not achieve baseline separation, the peak area was defined by the valley of the overlapping peaks, extending approximately 30 s forward or backward from the demarcation point. Therefore, the quantification of each glycopeptide was achieved by the peak areas of each glycopeptide MS spectra (Supplementary Data File 4).

## Data Availability

Data associated with the manuscript has been deposited within MassIVE repository “MSV000095017” and can additionally be accessed via ftp://massive.ucsd.edu/v08/MSV000095017/ or by logging in with the username “MSV000095017” and password “a”.

## Supporting Information Available

HCDpdEThcD fragmentation of IgG1 glycopeptides, quantitative glycopeptide isomer characterization, MS/MS-based characterization of by HILIC chromatography separated isobaric glycopeptides, annotation of the gray chromatographic peaks, quantitative glycan profiles, relative abundances of IgG subclasses, variation at the amino acid level between IgG allotypes within the IgG1, IgG2, IgG3, and IgG4 subclasses (PDF)
FASTA data (PDF)
Glycopeptides from IgG subclasses and their *m*/*z* value used in MS/MS method (XLSX)
Glycan compositions and glycan database used in the Byonic searches (XLSX)
Peak areas of glycopeptides obtained using Skyline (XLSX)
Transparent Peer Review report available (PDF)

## References

[ref1] Vidarsson G.; Dekkers G.; Rispens T. IgG subclasses and allotypes: from structure to effector functions. Front Immunol 2014, 5, 520 10.3389/fimmu.2014.00520. 25368619 PMC4202688

[ref2] Heiner D. C. Significance of immunoglobulin G subclasses. Am. J. Med. 1984, 76 (3a), 1 10.1016/0002-9343(84)90313-9. 6369973

[ref3] Jefferis R. Isotype and glycoform selection for antibody therapeutics. Arch. Biochem. Biophys. 2012, 526 (2), 159 10.1016/j.abb.2012.03.021. 22465822

[ref4] Liu S.; Liu X. IgG N-glycans. Adv. Clin Chem. 2021, 105, 1 10.1016/bs.acc.2021.02.001. 34809825

[ref5] de Taeye S. W.; Bentlage A. E. H.; Mebius M. M.; Meesters J. I.; Lissenberg-Thunnissen S.; Falck D.; Sénard T.; Salehi N.; Wuhrer M.; Schuurman J.; Labrijn A. F.; Rispens T.; Vidarsson G. FcγR Binding and ADCC Activity of Human IgG Allotypes. Front Immunol 2020, 11, 740 10.3389/fimmu.2020.00740. 32435243 PMC7218058

[ref6] Jungi T. W.; Brcic M.; Kuhnert P.; Spycher M. O.; Li F.; Nydegger U. E. Effect of IgG for intravenous use on Fc receptor-mediated phagocytosis by human monocytes. Clin. Exp. Immunol. 2008, 82 (1), 163 10.1111/j.1365-2249.1990.tb05421.x. PMC1535175 2208790

[ref7] Díaz de León J. S. A.; Aguilar I.; Barb A. W. Macrophage N-glycan processing inhibits antibody-dependent cellular phagocytosis. Glycobiology 2023, 33 (12), 1182–1192. 10.1093/glycob/cwad078. 37792857 PMC10876040

[ref8] Bernth Jensen J. M.; Laursen N. S.; Jensen R. K.; Andersen G. R.; Jensenius J. C.; Sørensen U. B. S.; Thiel S. Complement activation by human IgG antibodies to galactose-α-1,3-galactose. Immunology 2020, 161 (1), 66 10.1111/imm.13229. 32583419 PMC7450175

[ref9] Raju T. S. Terminal sugars of Fc glycans influence antibody effector functions of IgGs. Curr. Opin Immunol 2008, 20 (4), 471 10.1016/j.coi.2008.06.007. 18606225

[ref10] Chung A. W.; Crispin M.; Pritchard L.; Robinson H.; Gorny M. K.; Yu X.; Bailey-Kellogg C.; Ackerman M. E.; Scanlan C.; Zolla-Pazner S.; Alter G. Identification of antibody glycosylation structures that predict monoclonal antibody Fc-effector function. Aids 2014, 28 (17), 2523 10.1097/QAD.0000000000000444. 25160934 PMC4429604

[ref11] Lofano G.; Gorman M. J.; Yousif A. S.; Yu W.-H.; Fox J. M.; Dugast A.-S.; Ackerman M. E.; Suscovich T. J.; Weiner J.; Barouch D.; Streeck H.; Little S.; Smith D.; Richman D.; Lauffenburger D.; Walker B. D.; Diamond M. S.; Alter G. Antigen-specific antibody Fc glycosylation enhances humoral immunity via the recruitment of complement. Science Immunology 2018, 3 (26), eaat7796 10.1126/sciimmunol.aat7796. 30120121 PMC6298214

[ref12] Liu L. Antibody glycosylation and its impact on the pharmacokinetics and pharmacodynamics of monoclonal antibodies and Fc-fusion proteins. J. Pharm. Sci. 2015, 104 (6), 1866 10.1002/jps.24444. 25872915

[ref13] Yu X.; Marshall M. J. E.; Cragg M. S.; Crispin M. Improving Antibody-Based Cancer Therapeutics Through Glycan Engineering. BioDrugs 2017, 31 (3), 151 10.1007/s40259-017-0223-8. 28466278

[ref14] Shen Y.; You Y.; Xiao K.; Chen Y.; Tian Z. Large-Scale Identification and Fragmentation Pathways Analysis of N-Glycans from Mouse Brain. J. Am. Soc. Mass Spectrom. 2019, 30 (7), 1254 10.1007/s13361-019-02181-y. 31098956

[ref15] Liu M.; Huang L. Z. X.; Smits A. A.; Büll C.; Narimatsu Y.; van Kuppeveld F. J. M.; Clausen H.; de Haan C. A. M.; de Vries E. Human-type sialic acid receptors contribute to avian influenza A virus binding and entry by hetero-multivalent interactions. Nat. Commun. 2022, 13 (1), 4054 10.1038/s41467-022-31840-0. 35831293 PMC9279479

[ref16] Anthony R. M.; Nimmerjahn F.; Ashline D. J.; Reinhold V. N.; Paulson J. C.; Ravetch J. V. Recapitulation of IVIG Anti-Inflammatory Activity with a Recombinant IgG Fc. Science 2008, 320 (5874), 373 10.1126/science.1154315. 18420934 PMC2409116

[ref17] Wang T.; Hoi K. M.; Stöckmann H.; Wan C.; Sim L. C.; Shi Jie Tay N.; Poo C. H.; Woen S.; Yang Y.; Zhang P.; Rudd P. M. LC/MS-based Intact IgG and Released Glycan Analysis for Bioprocessing Applications. Biotechnol J. 2018, 13 (4), e1700185 10.1002/biot.201700185. 29341427

[ref18] Zhou X.; Song W.; Novotny M. V.; Jacobson S. C. Fractionation and characterization of sialyl linkage isomers of serum N-glycans by CE-MS. J. Sep Sci. 2022, 45 (17), 3348 10.1002/jssc.202200223. 35819141 PMC9473921

[ref19] de Haan N.; Reiding K. R.; Haberger M.; Reusch D.; Falck D.; Wuhrer M. Linkage-specific sialic acid derivatization for MALDI-TOF-MS profiling of IgG glycopeptides. Anal. Chem. 2015, 87 (16), 8284 10.1021/acs.analchem.5b02426. 26191964

[ref20] Zou Y.; Hu J.; Jie J.; Lai J.; Li M.; Liu Z.; Zou X. Comprehensive analysis of human IgG Fc N-glycopeptides and construction of a screening model for colorectal cancer. J. Proteomics 2020, 213, 103616 10.1016/j.jprot.2019.103616. 31846768

[ref21] Meng X.; Wang F.; Gao X.; Wang B.; Xu X.; Wang Y.; Wang W.; Zeng Q. Association of IgG N-glycomics with prevalent and incident type 2 diabetes mellitus from the paradigm of predictive, preventive, and personalized medicine standpoint. Epma j 2023, 14 (1), 1 10.1007/s13167-022-00311-3. PMC9971369 36866157

[ref22] Zhang Z. J.; Wang H. F.; Lian T. Y.; Zhou Y. P.; Xu X. Q.; Guo F.; Wei Y. P.; Li J. Y.; Sun K.; Liu C.; Pan L. R.; Ren M.; Nie L.; Dai H. L.; Jing Z. C. Human Plasma IgG N-Glycome Profiles Reveal a Proinflammatory Phenotype in Chronic Thromboembolic Pulmonary Hypertension. Hypertension 2023, 80 (9), 1929 10.1161/HYPERTENSIONAHA.123.21408. 37449418

[ref23] Chandler K. B.; Mehta N.; Leon D. R.; Suscovich T. J.; Alter G.; Costello C. E. Multi-isotype Glycoproteomic Characterization of Serum Antibody Heavy Chains Reveals Isotype- and Subclass-Specific N-Glycosylation Profiles. Mol. Cell Proteomics 2019, 18 (4), 686 10.1074/mcp.RA118.001185. 30659065 PMC6442369

[ref24] Blöchl C.; Regl C.; Huber C. G.; Winter P.; Weiss R.; Wohlschlager T. Towards middle-up analysis of polyclonal antibodies: subclass-specific N-glycosylation profiling of murine immunoglobulin G (IgG) by means of HPLC-MS. Sci. Rep. 2020, 10 (1), 18080 10.1038/s41598-020-75045-1. 33093535 PMC7581757

[ref25] Molnarova K.; Cokrtova K.; Tomnikova A.; Krizek T.; Kozlik P. Liquid chromatography and capillary electrophoresis in glycomic and glycoproteomic analysis. Monatsh. Chem. 2022, 153 (9), 659 10.1007/s00706-022-02938-4. 35754790 PMC9212196

[ref26] Molnarova K.; Kozlík P. Comparison of Different HILIC Stationary Phases in the Separation of Hemopexin and Immunoglobulin G Glycopeptides and Their Isomers. Molecules 2020, 25 (20), 4655 10.3390/molecules25204655. 33065988 PMC7594091

[ref27] Maliepaard J. C. L.; Damen J. M. A.; Boons G. P. H.; Reiding K. R. Glycoproteomics-Compatible MS/MS-Based Quantification of Glycopeptide Isomers. Anal. Chem. 2023, 95 (25), 9605 10.1021/acs.analchem.3c01319. 37319314 PMC10308332

[ref28] Lauc G.; Huffman J. E.; Pučić M.; Zgaga L.; Adamczyk B.; Mužinić A.; Novokmet M.; Polašek O.; Gornik O.; Krištić J.; Keser T.; Vitart V.; Scheijen B.; Uh H. W.; Molokhia M.; Patrick A. L.; McKeigue P.; Kolčić L.; Lukić L. K.; Swann O.; van Leeuwen F. N.; Ruhaak L. R.; Houwing-Duistermaat J. J.; Slagboom P. E.; Beekman M.; de Craen A. J. M.; Deelder A. M.; Zeng Q.; Wang W.; Hastie N. D.; Gyllensten U.; Wilson J. F.; Wuhrer M.; Wright A. F.; Rudd P. M.; Hayward C.; Aulchenko Y.; Campbell H.; Rudan I. Loci associated with N-glycosylation of human immunoglobulin G show pleiotropy with autoimmune diseases and haematological cancers. PLoS Genet 2013, 9 (1), e1003225 10.1371/journal.pgen.1003225. 23382691 PMC3561084

[ref29] Blundell P. A.; Lu D.; Dell A.; Haslam S.; Pleass R. J. Choice of Host Cell Line Is Essential for the Functional Glycosylation of the Fc Region of Human IgG1 Inhibitors of Influenza B Viruses. J. Immunol 2020, 204 (4), 1022 10.4049/jimmunol.1901145. 31907284 PMC6994840

[ref30] Uhler R.; Popa-Wagner R.; Kröning M.; Brehm A.; Rennert P.; Seifried A.; Peschke M.; Krieger M.; Kohla G.; Kannicht C.; Wiedemann P.; Hafner M.; Rosenlöcher J. Glyco-engineered HEK 293-F cell lines for the production of therapeutic glycoproteins with human N-glycosylation and improved pharmacokinetics. Glycobiology 2021, 31 (7), 859 10.1093/glycob/cwaa119. 33403396

[ref31] Napodano C.; Marino M.; Stefanile A.; Pocino K.; Scatena R.; Gulli F.; Rapaccini G. L.; Delli Noci S.; Capozio G.; Rigante D.; Basile U. Immunological Role of IgG Subclasses. Immunol Invest 2021, 50 (4), 427 10.1080/08820139.2020.1775643. 32522062

[ref32] Lu G.; Holland L. A. Profiling the N-Glycan Composition of IgG with Lectins and Capillary Nanogel Electrophoresis. Anal. Chem. 2019, 91 (2), 1375 10.1021/acs.analchem.8b03725. 30525457 PMC6335613

[ref33] Shkunnikova S.; Mijakovac A.; Sironic L.; Hanic M.; Lauc G.; Kavur M. M. IgG glycans in health and disease: Prediction, intervention, prognosis, and therapy. Biotechnol Adv. 2023, 67, 108169 10.1016/j.biotechadv.2023.108169. 37207876

[ref34] Haslund-Gourley B. S.; Wigdahl B.; Comunale M. A. IgG N-glycan Signatures as Potential Diagnostic and Prognostic Biomarkers. Diagnostics (Basel) 2023, 13 (6), 1016 10.3390/diagnostics13061016. 36980324 PMC10047871

[ref35] Bondt A.; Hoek M.; Tamara S.; de Graaf B.; Peng W.; Schulte D.; van Rijswijck D. M. H.; den Boer M. A.; Greisch J. F.; Varkila M. R. J.; et al. Human plasma IgG1 repertoires are simple, unique, and dynamic. Cell Syst 2021, 12 (12), 1131 10.1016/j.cels.2021.08.008. 34613904 PMC8691384

[ref36] Foss S.; Sakya S. A.; Aguinagalde L.; Lustig M.; Shaughnessy J.; Cruz A. R.; Scheepmaker L.; Mathiesen L.; Ruso-Julve F.; Anthi A. K.; Gjølberg T. T.; Mester S.; Bern M.; Evers M.; Bratlie D. B.; Michaelsen T. E.; Schlothauer T.; Sok D.; Bhattacharya J.; Leusen J.; Valerius T.; Ram S.; Rooijakkers S. H. M.; Sandlie I.; Andersen J. T. Human IgG Fc-engineering for enhanced plasma half-life, mucosal distribution and killing of cancer cells and bacteria. Nat. Commun. 2024, 15 (1), 2007 10.1038/s41467-024-46321-9. 38453922 PMC10920689

[ref37] de Taeye S. W.; Rispens T.; Vidarsson G. The Ligands for Human IgG and Their Effector Functions. Antibodies 2019, 8 (2), 30 10.3390/antib8020030. 31544836 PMC6640714

