# Supplementary material for: Human IgG Subclasses Differ in the Structural Elements of Their N-Glycosylation
Source: ACS Cent Sci. 2024 Oct 10;10(11):2048–58. doi: 10.1021/acscentsci.4c01157 (PMC11613209; doi:10.1021/acscentsci.4c01157)
Supplement: Supplementary file 1 — oc4c01157_si_001.pdf [file oc4c01157_si_001.pdf]

Supplementary Material

Human IgG subclasses differ in the structural elements of their *N*-glycosylation

Weiwei Wang[1,2,3,*], Joshua C. L. Maliepaard[1,2], Timon Damelang[1,5,6], Gestur Vidarsson[1,2,4], Albert J.R. Heck[1,2], Karli R. Reiding[1,2,*]

[1] Biomolecular Mass Spectrometry and Proteomics, Bijvoet Center for Biomolecular Research and Utrecht Institute for Pharmaceutical Sciences, Utrecht University, Padualaan 8, 3584 CH Utrecht, The Netherlands.

[2] Netherlands Proteomics Center, Utrecht, The Netherlands.

[3] School of Pharmaceutical Science, Shanghai Jiao Tong University, 800 Dongchuan Road, Shanghai, People's Republic of China

[4] Sanquin Research, Immunoglobulin Research Laboratory, Amsterdam, The Netherlands.

[5] Sanquin Research, Department of Experimental Immunohematology and Landsteiner Laboratory, Amsterdam, the Netherlands.

[6] Sanquin Research, Department of Immunopathology, Amsterdam, the Netherlands.

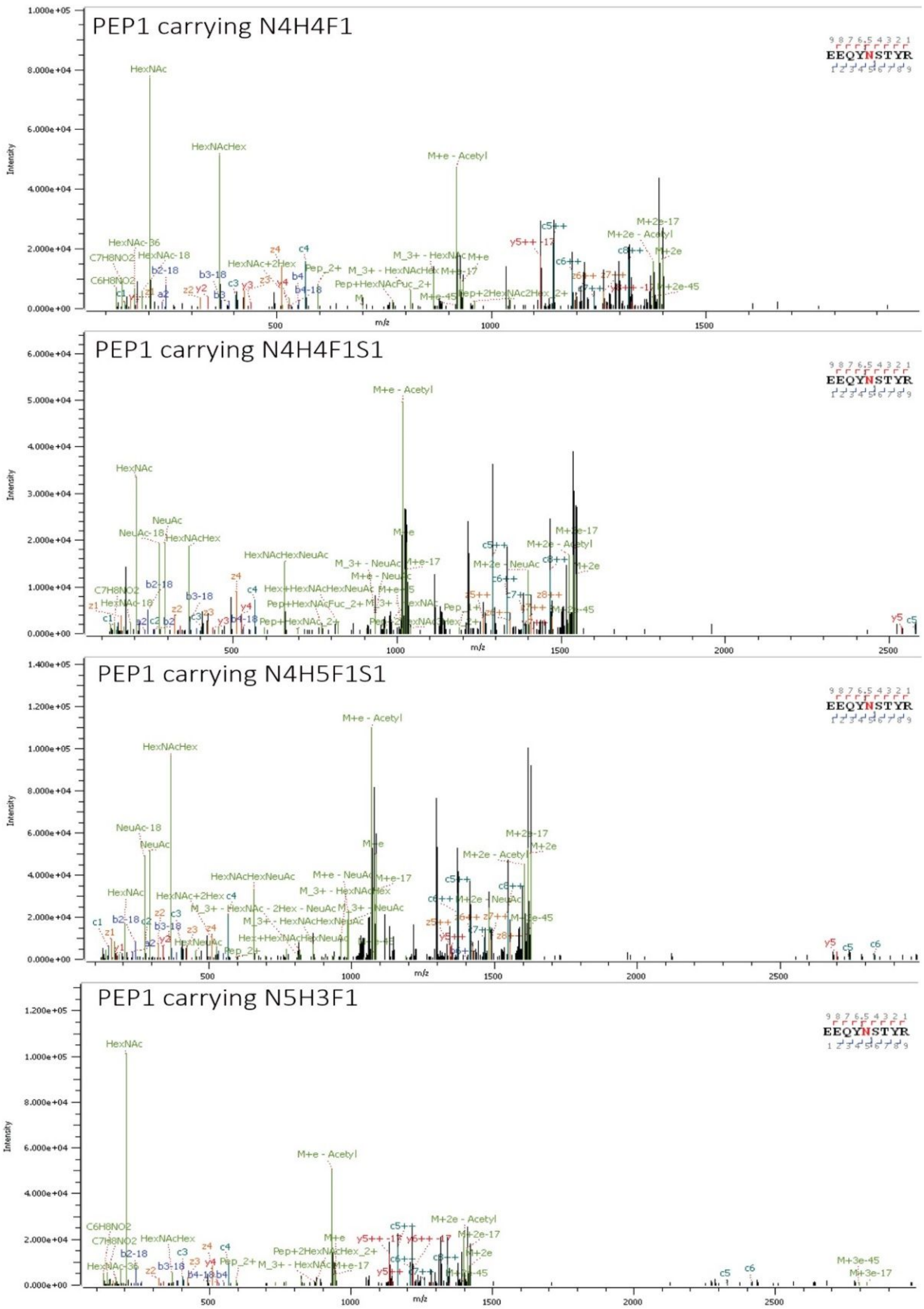

Figure S1. HCDpdEThcD fragmentation of IgG1 glycopeptides. Demonstrated here are PEP1 (sequence EEQYNSTYR) carrying glycans N4H4F1, N4H4F1S1, N4H5F1S1, and N5H3F1. As can be seen, a comprehensive range of b/y and c/z ions informs on the peptide identity.

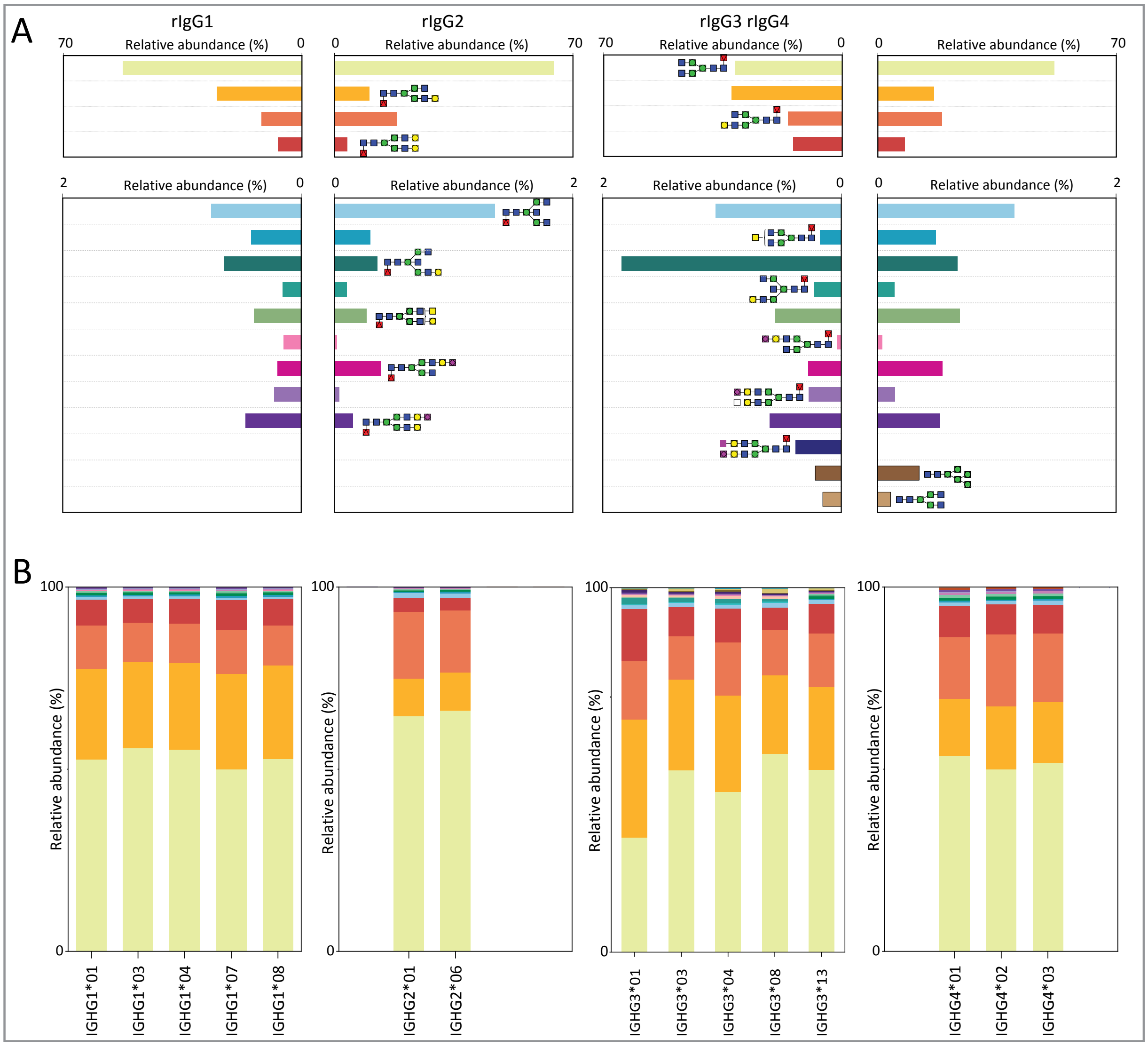

Figure S2. Quantitative glycopeptide isomer characterization as occurring on the Fc domains from distinct rIgG subclasses and allotypes. A) Relative abundance of observed glycan structures within the Fc domains of distinct rIgG subclasses. The top panels depict the most abundant glycan structures, while the bottom panel shows glycan structures that contribute maximally 2% to the full glycan composition (note the different scales). B) Comprehensive glycan compositions within the Fc domains from rIgG subclasses and allotypes. For each subclass of IgG several allotypes were studied, for IgG1 5, for IgG2 2, for IgG3 5 and for IgG4 3. The terminology for the allotype originates from Table S1.

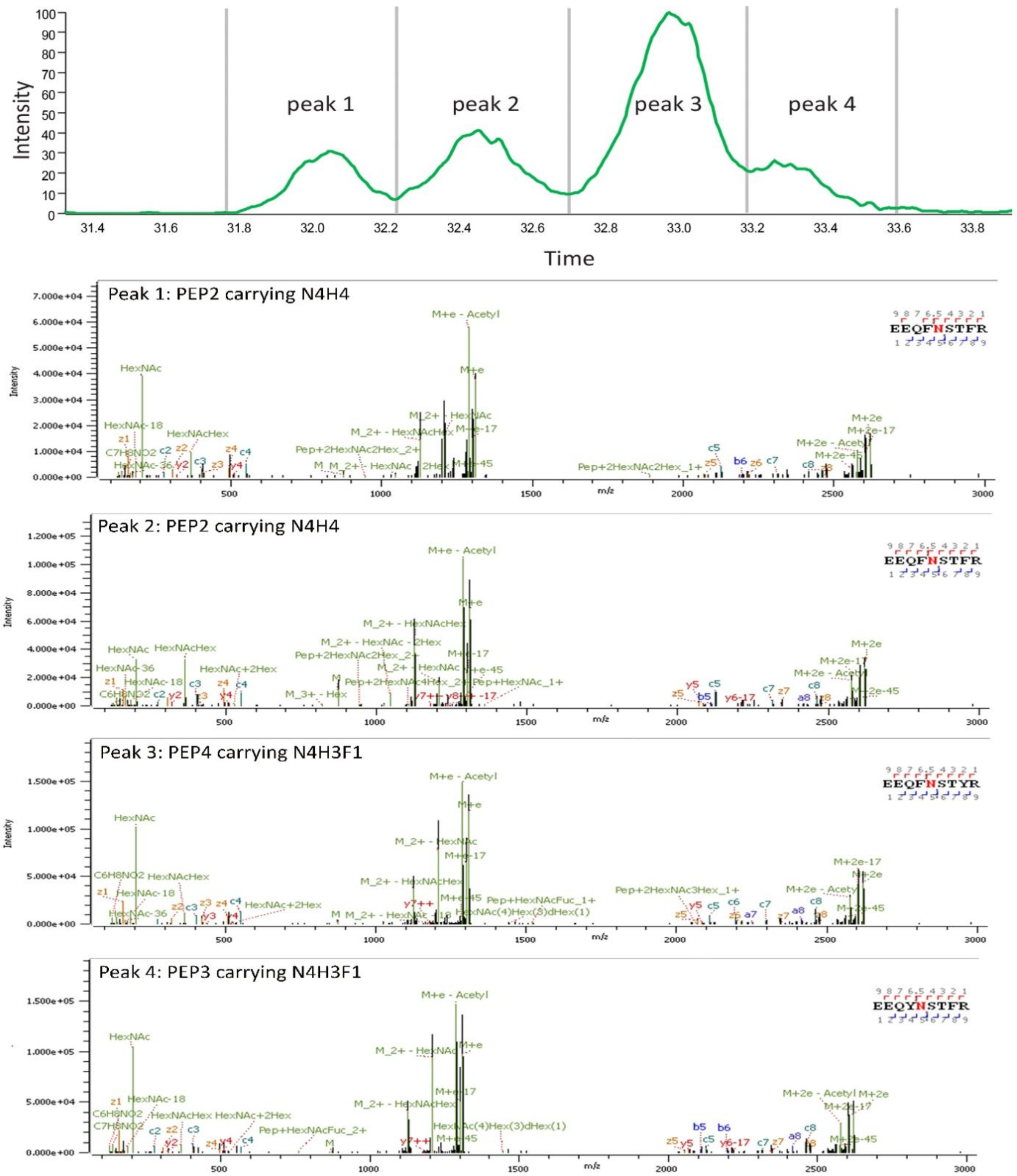

Figure S3. MS/MS-based characterization of by HILIC chromatography separated isobaric glycopeptides (Mass=2617.04) from IgG subclasses in human plasma. Initially, four distinct chromatographic peaks within a narrow mass range were observed. Subsequent MS/MS analysis revealed the differences in the peptide sequences among these chromatographic peaks. The first and second peaks were identified as IgG2 glycopeptides carrying N4H4, the third peak as an IgG4 glycopeptide carrying N4H3F1, and the fourth as an IgG3 glycopeptide carrying N4H3F1.

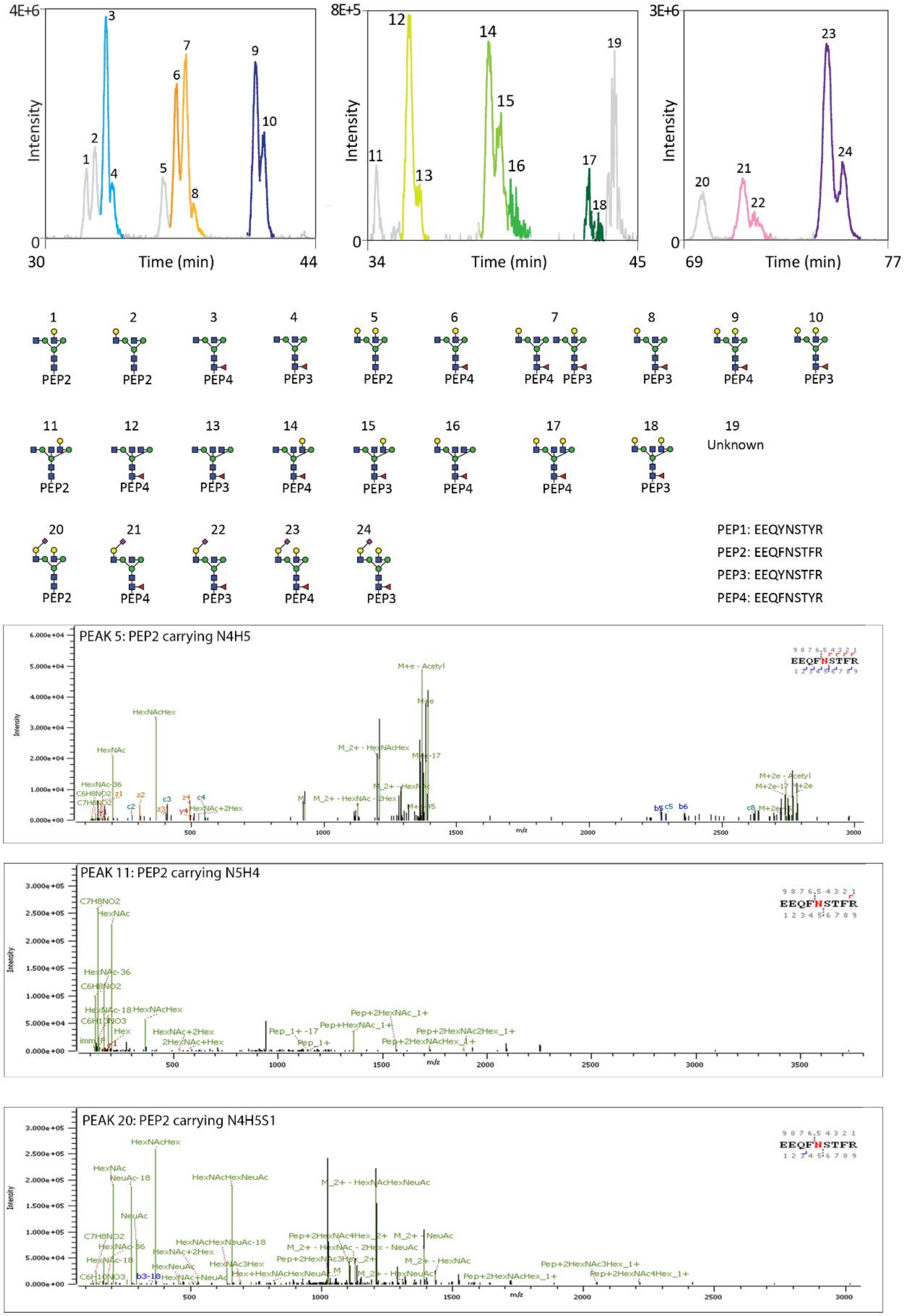

4E+6
Intensity
0
30
Time (min)
44
8E+5
Intensity
0
34
Time (min)
45
3E+6
Intensity
0
69
Time (min)
77
19
Unknown
PEP1: EEQYNSTYR
PEP2: EEQFNSTFR
PEP3: EEQYNSTFR
PEP4: EEQFNSTYR
PEAK 5: PEP2 carrying N4H5
PEAK 11: PEP2 carrying N5H4
PEAK 20: PEP2 carrying N4H5S1
EEQFNSTFR

Figure S4. The annotation of the gray chromatographic peaks observed in Figure 4. All chromatographic peaks have been numerically labelled and the corresponding glycopeptides with specific glycan structures are displayed. The MS2 spectra for identifiable gray chromatographic peaks are also shown. All peak assignments are based on their corresponding MS2 fragmentation data and our knowledge of the retention behavior of glycopeptides in HILIC chromatography. Peaks 1-4 share the same mass; their assignments can be referenced in Figure S2. Peaks 5-8 also share the same mass, with Peak 5 identified as PEP2 carrying N4H5. Peaks 6 and 7 are derived from PEP4 and PEP3, each carrying N4H4F1. Peaks 11-13 share the same mass; Peak 11 is PEP2 carrying N5H4, while Peaks 12 and 13 are from PEP4 and PEP3, respectively, each carrying N5H3F1. Peak 19 is unidentified but, given its consistent presence in human blood samples, is believed to be another glycopeptide from the blood. Peaks 20-22 share the same mass, with Peak 20 identified as PEP2 carrying 3-branched N4H5S1, and Peaks 21 and 22 from PEP4 and PEP3, respectively, each carrying N4H4F1S1.

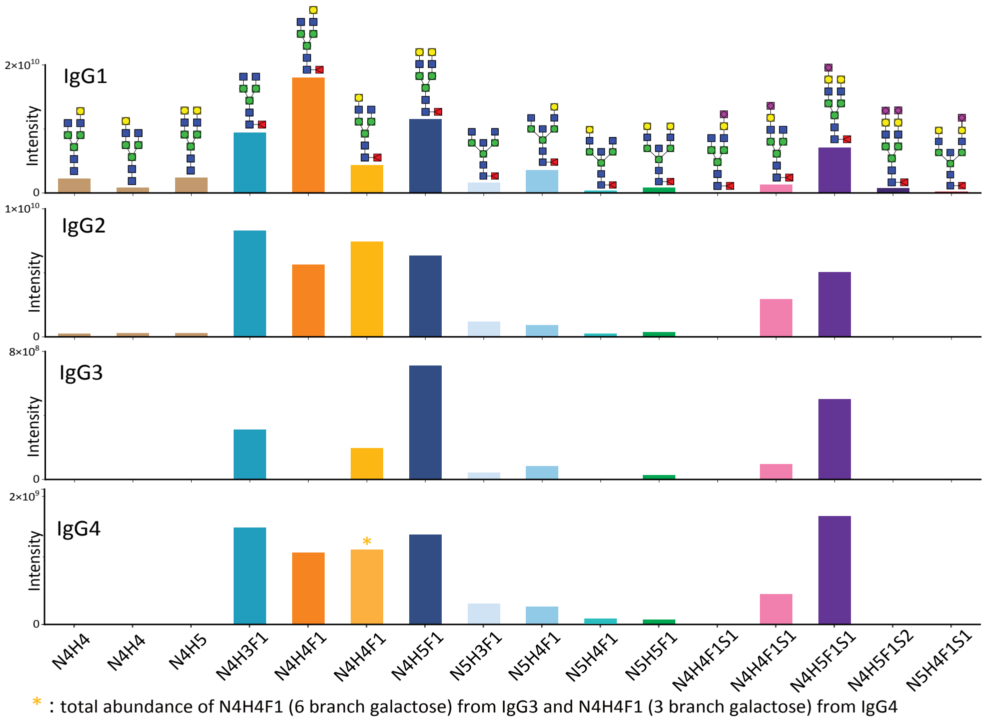

Figure S5. Quantitative glycan profiles as observed for each of the IgG subclasses as present in pooled human plasma. In the observed glycan structure intensities across the four IgG subclasses, the total intensities reveal a hierarchy where IgG1 surpasses IgG2, which in turn is higher than IgG4 and IgG3. A common characteristic of the glycan profiles across all subclasses is the predominance of core fucosylated glycans. Notably, the subclasses consistently exhibit a higher abundance of N4H3F1, N4H4F1, N4H5F1, N4H4F1S1, and N4H5F1S1 structures. Other glycan types are present at lower abundances.

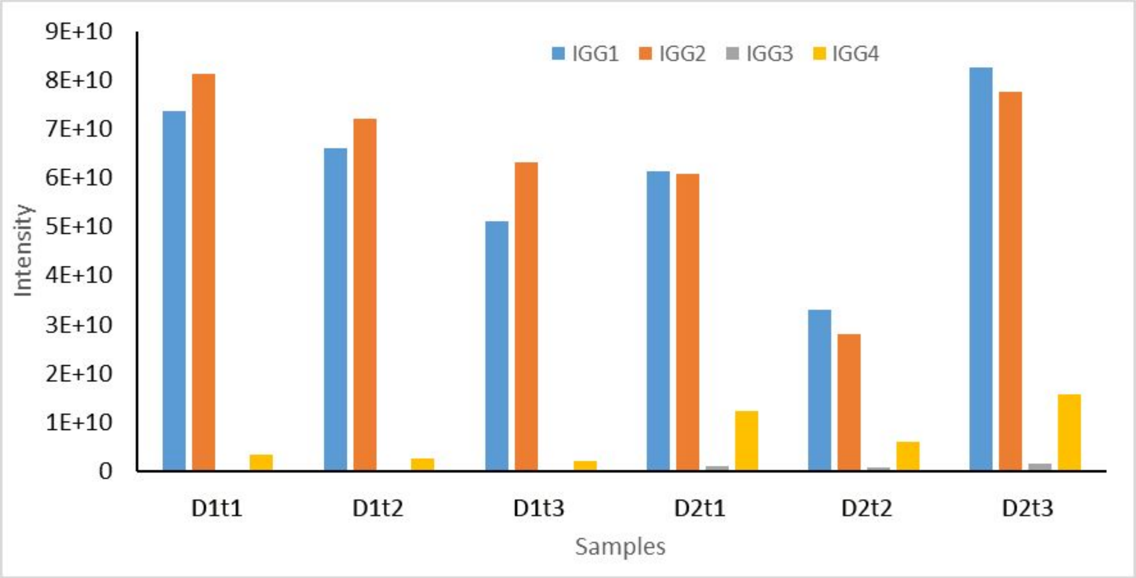

Figure S6. The relative abundances of IgG subclasses as quantified by their glycopeptides. Samples were collected at three distinct time points, displaying some variation in between donors and in time. Glycopeptide areas were integrated from the raw MS signal using Skyline, whereby all peptide glycoforms were summed to the displayed intensity.

Table S1. Variation at the amino acid level between IgG allotypes within the IgG1, IgG2, IgG3, and IgG4 subclasses. For each domain, CH1, hinge, CH2 and CH3, amino acid differences between polymorphic variants are indicated with specific colors. Polymorphisms in the hinge region are identified by the presence or absence of hinge exons (A and B). These allotypes are adapted from Vidarsson et al 2020[1] .

IgG heavy chain domain	CH1					Hinge					CH2						CH3												
IMGT exon numbering	59	72	75	76	97	Exons				Length	52	61	62	66	79	109	16	18	38	39	44	52	57	69	79	82	91	95	96
EU numbering	176	189	192	193	214	A	B	B	B		282	291	292	296	309	339	356	358	378	379	384	392	397	409	419	422	431	435	436
IgG1 allotypes																													
IGHG1*03	S	P	S	L	R	+				15	V	P	R	Y	L	A	E	M	A	V	N	K	V	K	Q	V	A	H	Y
IGHG1*08	S	P	S	L	R	+				15	V	P	R	Y	L	A	D	L	A	V	N	K	V	K	Q	V	A	H	Y
IGHG1*01	S	P	S	L	K	+				15	V	P	R	Y	L	A	D	L	A	V	N	K	V	K	Q	V	A	H	Y
IGHG1*07	S	P	S	L	K	+				15	V	P	R	Y	L	A	D	L	A	V	N	K	V	K	Q	V	G	H	Y
IGHG1*04	S	P	S	L	K	+				15	V	P	R	Y	L	A	D	L	A	V	N	K	V	K	Q	I	A	H	Y
IgG2 allotypes																													
IGHG2*01	S	P	N	F	T	+				12	V	P	R	F	V	T	E	M	A	V	N	K	M	K	Q	V	A	H	Y
IGHG2*06	S	P	N	F	T	+				12	V	P	R	F	V	T	E	M	S	V	N	K	M	K	Q	V	A	H	Y
IgG3 allotypes																													
IGHG3*01	S	P	S	L	R	+	+	+	+	62	V	P	R	Y	L	T	E	M	A	V	S	N	M	K	Q	I	A	R	F
IGHG3*04	S	P	S	L	R	+	–	–	+	32	V	P	R	Y	L	T	E	M	A	V	S	N	M	K	Q	I	A	R	F
IGHG3*08	S	P	S	L	R	+	+	+	+	62	V	P	R	Y	L	T	E	M	A	V	N	N	M	K	Q	I	A	R	F
IGHG3*13	S	P	S	L	R	+	+	+	+	62	V	P	R	Y	L	T	E	M	A	V	S	K	M	K	E	I	A	R	F
IGHG3*03	S	P	S	L	R	+	–	+	+	47	V	P	R	Y	L	T	E	M	A	V	S	N	V	K	Q	V	A	R	F
IgG4 allotypes																													
IGHG4*01	S	P	S	L	R	+				12	V	P	R	F	L	A	E	M	A	V	N	K	V	R	E	V	A	H	Y
IGHG4*03	S	P	S	L	R	+				12	V	P	R	F	L	A	E	M	A	V	N	K	V	K	E	V	A	H	Y
IGHG4*02	S	P	S	L	R	+				12	V	P	R	F	V	A	E	M	A	V	N	K	V	R	E	V	A	H	Y

Reference

(1) de Taeye, S. W.; Bentlage, A. E. H.; Mebius, M. M.; Meesters, J. I.; Lissenberg-Thunnissen, S.; Falck, D.; Sénard, T.; Salehi, N.; Wuhrer, M.; Schuurman, J., Labrijn, A. F., Rispens, T., Vidarsson, G. FcγR Binding and ADCC Activity of Human IgG Allotypes. *Front Immunol* **2020,** *11*, 740, DOI: 10.3389/fimmu.2020.00740
