# Supplementary material for: Human IgG Subclasses Differ in the Structural Elements of Their N-Glycosylation
Source: ACS Cent Sci. 2024 Oct 10;10(11):2048–58. doi: 10.1021/acscentsci.4c01157 (PMC11613209; doi:10.1021/acscentsci.4c01157)
Supplement: Supplementary file 2 — oc4c01157_si_002.pdf [file oc4c01157_si_002.pdf]

>IGHG1
ASTKGPSVFPLAPSSKSTSGGTAALGCLVKDYFPEPVTVSWNSGALTSGVHTFPAVLQSS
GLYSLSSVVTVPSSSLGTQT
YICNVNHKPSNTKVDKKVEPKSCDKTHTCPPCPAPELLGGPSVFLFPPKPKDTLMISRTP
EVTCVVVDVSHEDPEVKFNW
YVDGVEVHNAKTKPREEQYNSTYRVVSVLTVLHQDWLNGKEYKCKVSNKALPAPIEK
TISKAKGQPREPQVYTLPPSRDE
LTKNQVSLTCLVKGFYPSDIAVEWESNGQPENNYKTTPPVLDSDGSFFLYSKLTVDKSR
WQQGNVFSCSVMHEALHNHYT
QKSLSLSPGK
>IGHG2
ASTKGPSVFPLAPCSRSTSESTAALGCLVKDYFPEPVTVSWNSGALTSGVHTFPAVLQSS
GLYSLSSVVTVPSSNFGTQT
YTCNVDHKPSNTKVDKTVERKCCVECPPCPAPPVAGPSVFLFPPKPKDTLMISRTPEVTC
VVVDVSHEDPEVQFNWYVDG
VEVHNAKTKPREEQFNSTFRVVSVLTVVHQDWLNGKEYKCKVSNKGLPAPIEKTISKTK
GQPREPQVYTLPPSREEMTKN
QVSLTCLVKGFYPSDIAVEWESNGQPENNYKTTPPMLDSDGSFFLYSKLTVDKSRWQQ
GNVFSCSVMHEALHNHYTQKSL
SLSPGK
>IGHG3
ASTKGPSVFPLAPCSRSTSGGTAALGCLVKDYFPEPVTVSWNSGALTSGVHTFPAVLQSS
GLYSLSSVVTVPSSSLGTQT
YTCNVNHKPSNTKVDKRVELKTPLGDTTHTCPRCPEPKSCDTPPPCPRCPEPKSCDTPPP
CPRCPEPKSCDTPPPCPRCP
APELLGGPSVFLFPPKPKDTLMISRTPEVTCVVVDVSHEDPEVQFKWYVDGVEVHNAKT
KPREEQYNSTFRVVSVLTVLH
QDWLNGKEYKCKVSNKALPAPIEKTISKTKGQPREPQVYTLPPSREEMTKNQVSLTCLV
KGFYPSDIAVEWESSGQPENN
YNTTPPMLDSDGSFFLYSKLTVDKSRWQQGNIFSCSVMHEALHNRFTQKSLSLSPGK
>IGHG4
ASTKGPSVFPLAPCSRSTSESTAALGCLVKDYFPEPVTVSWNSGALTSGVHTFPAVLQSS
GLYSLSSVVTVPSSSLGTKT
YTCNVDHKPSNTKVDKRVESKYGPPCPSCPAPEFLGGPSVFLFPPKPKDTLMISRTPEVT
CVVVDVSQEDPEVQFNWYVD
GVEVHNAKTKPREEQFNSTYRVVSVLTVLHQDWLNGKEYKCKVSNKGLPSSIEKTISKA
KGQPREPQVYTLPPSQEEMTK
NQVSLTCLVKGFYPSDIAVEWESNGQPENNYKTTPPVLDSDGSFFLYSRLTVDKSRWQE
GNVFSCSVMHEALHNHYTQKS
LSLSLGK
