# Supplementary material for: Human IgG Subclasses Differ in the Structural Elements of Their N-Glycosylation
Source: ACS Cent Sci. 2024 Oct 10;10(11):2048–58. doi: 10.1021/acscentsci.4c01157 (PMC11613209; doi:10.1021/acscentsci.4c01157)
Supplement: Supplementary file 6 — oc4c01157_si_006.pdf [file oc4c01157_si_006.pdf]

oc-2024-01157n.R1

Name: Peer Review Information for "Human IgG subclasses differ in the structural elements of their <i>N</i>-glycosylation"

First Round of Reviewer Comments

Reviewer: 1

Comments to the Author

Wang et al. present a highly interesting study using HILIC LC and targeted glycoproteomics with low-collision-energy HCD to structurally characterize N-glycopeptides derived from IgG1-4 subclasses. They show that recombinantly expressed (from HEK293) IgG subclasses consistently display different structural characteristics at the conserved N-glycosite, but that allotypes exhibit nearly identical structural characteristics within a subclass. They also analyze IgG glycopeptides from human plasma (two donors) to show that plasma IgG subclass glycoprofiles differ from each other and from recombinant versions, especially with diminished glycan diversity in plasma vs recombinant. They also show temporal stability of glycan structural differences across IgG subtypes within a given donor. This is interesting and important work toward advancing structural glycoproteomics, and I see this manuscript as largely ready for publication as is. I would ask the authors address the following minor comments through brief discussion in the text prior to publication.

1. Figure 4 shows extracted ion chromatograms for various isobaric glycopeptide species, and in some panels, there are significant contributions to the XICs that are grayed out instead of a color of the indicated glycopeptide species. What are those grayed out species? Are they the isobaric IgG3, IgG4, and IgG2 glycopeptides discussed on page 7 and in Figure S2, or are there other isobaric species in plasma that can confound data analysis if experimentalists are not careful? Clarifying this in the text would be helpful.

2. I am surprised no di-sialylated N-glycans were observed or discussed in this work. Could the authors comment in the discussion on why they were not present in the samples or focused on for this work? If they are present in the samples, I think they should be included and discussed in this manuscript because these glycoforms are interesting and likely contribute to the conclusions about IgG subtype profiles that the authors focus on for this study. If they are not present, that is interesting (perhaps confusing?) and should be noted for readers.

Reviewer: 2

Comments to the Author

In the manuscript by Wang et al., the authors investigate N-glycans on various IgG subtypes. The paper is well written, thorough, and the figures are great. I recommend publishing with the following minor edits:

- The synopsis should perhaps be expanded so as to be closer to the 60 word limit

- It might be useful to include b/y ions in Figure 2

- HILIC is mentioned throughout the text but is not included in the methods; please add

- Page 7, line 40, there's an additional "with" in the text

- Ion intensity in the methods is listed as "5 x 103" and I believe it should be 10^3?

Author's Response to Peer Review Comments:

We thank the editor and reviewers for their time and favorable assessment of our manuscript. We have addressed the reviewer comments as described point-by-point below. We hope the revised manuscript will be considered suitable for publication in ACS Central Science.

Reviewer(s)' Comments to Author:

Reviewer: 1

Recommendation: Publish in ACS Central Science after minor revisions noted.

Comments:

Wang et al. present a highly interesting study using HILIC LC and targeted glycoproteomics with low-collision-energy HCD to structurally characterize N-glycopeptides derived from IgG14 subclasses. They show that recombinantly expressed (from HEK293) IgG subclasses consistently display different structural characteristics at the conserved N-glycosite, but that allotypes exhibit nearly identical structural characteristics within a subclass. They also analyze IgG glycopeptides

from human plasma (two donors) to show that plasma IgG subclass glycoprofiles differ from each other and from recombinant versions, especially with diminished glycan diversity in plasma vs recombinant. They also show temporal stability of glycan structural differences across IgG subtypes within a given donor. This is interesting and important work toward advancing structural glycoproteomics, and I see this manuscript as largely ready for publication as is. I would ask the authors address the following minor comments through brief discussion in the text prior to publication.

1. Figure 4 shows extracted ion chromatograms for various isobaric glycopeptide species, and in some panels, there are significant contributions to the XICs that are grayed out instead of a color of the indicated glycopeptide species. What are those grayed out species? Are they the isobaric IgG3, IgG4, and IgG2 glycopeptides discussed on page 7 and in Figure S2, or are there other isobaric species in plasma that can confound data analysis if experimentalists are not careful?

Clarifying this in the text would be helpful.

Answer: Thank you for highlighting this concern. To address it, we have included Figure S4 in the Supporting Information, which elucidates the gray peaks observed in the chromatograms. By integrating MS1 data, MS2 outputs from Byonic, and our knowledge of the retention behavior of glycopeptides on HILIC, we conclude that most peaks are derived from glycopeptides of IgG subclasses, with one peak potentially originating from other components in human plasma. In detail, we have supplemented the supporting materials with the following content:

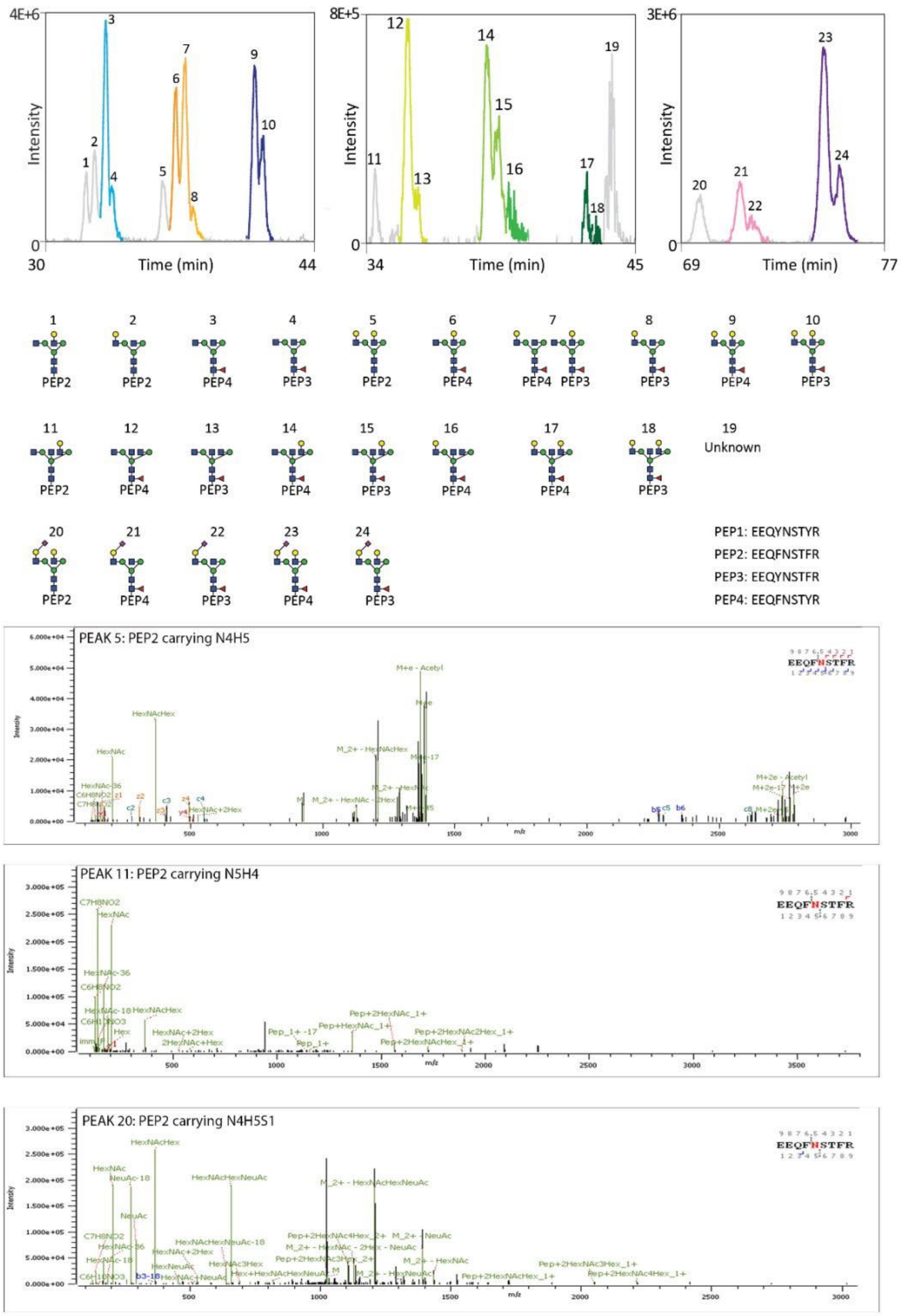

"Figure S4 The annotation of the gray chromatographic peaks observed in Figure 4. *All chromatographic peaks have been numerically labelled and the corresponding glycopeptides with specific glycan structures are displayed. The MS2 spectra for identifiable gray chromatographic peaks are also shown. All peak assignments are based on their corresponding MS2 fragmentation data and our knowledge of the retention behavior of glycopeptides in HILIC chromatography. Peaks 1-4 share the same mass; their assignments can be referenced in Figure S2. Peaks 5-8 also share the same mass, with Peak 5 identified as PEP2 carrying N4H5. Peaks 6 and 7 are derived*

from PEP4 and PEP3, each carrying N4H4F1. Peaks 11-13 share the same mass; Peak 11 is PEP2 carrying N5H4, while Peaks 12 and 13 are from PEP4 and PEP3, respectively, each carrying N5H3F1. Peak 19 is unidentified but, given its consistent presence in human blood samples, is believed to be another glycopeptide from the blood. Peaks 20-22 share the same mass, with Peak 20 identified as PEP2 carrying 3-branched N4H5S1, and Peaks 21 and 22 from PEP4 and PEP3, respectively, each carrying N4H4F1S1."

2. I am surprised no di-sialylated N-glycans were observed or discussed in this work. Could the authors comment in the discussion on why they were not present in the samples or focused on for this work? If they are present in the samples, I think they should be included and discussed in this manuscript because these glycoforms are interesting and likely contribute to the conclusions about IgG subtype profiles that the authors focus on for this study. If they are not present, that is interesting (perhaps confusing?) and should be noted for readers.

Answer: Thank you for your inquiry. To address your question, we re-evaluated the raw data. First, among the recombinant IgG subclasses, the abundance of *N*-glycans with sialic acid is notably low. We detected minimal occurrences of disialylated glycans in rIgG3, which comprised less than 0.5% of the total *N*-glycan content, as illustrated in Figure S1. In human plasma, considering the lower concentrations of IgG3 and IgG4, we specifically revisited the N4H5S2F1 in IgG1 and IgG2. Our findings indicate that disialylated glycopeptide signals are likely visible in MS1, but that MS2 quality is not sufficient to definitively prove this.

In detail, we conducted extracted ion chromatograms specifically for sialylated glycopeptides derived from PEP1 and PEP2 and annotated their glycan structures. As depicted in the figures below, these chromatograms, sourced from pooled human plasma samples D1T1 and D2T1, distinctly show PEP1 and PEP2 carrying N4H5F1S1 between 70-76 minutes. Notably, very small peaks potentially representing PEP1 or PEP2 carrying N4H5F1S2 were observed at 98-102 minutes. However, the lack of comprehensive MS2 data prevents definitive structural verification.

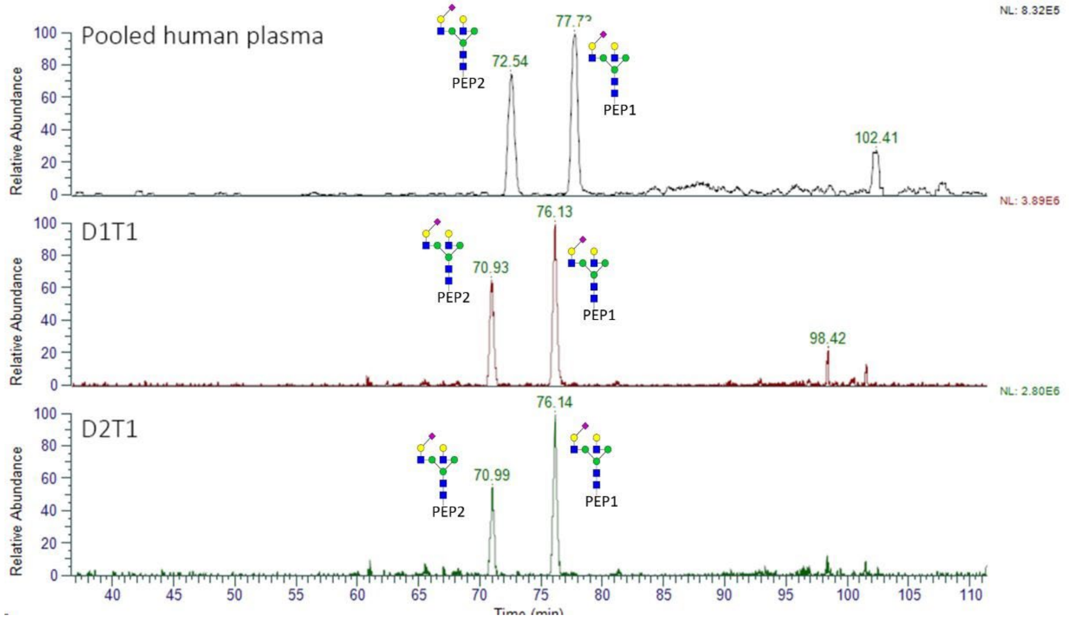

Figure Chromatograms of Sialylated Glycopeptides from IgG1 and IgG2 in Human Plasma

Based on our data and the significance of disialylation, we have made the following modifications to the manuscript:

"To recall, recombinant IgGs displayed high abundances of both 6-branch and 3-branch sialylation in their monosialylated glycans. MS1 signals potentially belonging to disialylated glycopeptides were detected in the human plasma as well, but their low signal intensity precluded the necessary MS2 quality to unambiguously make the determination."

Additional Questions:

Quality of experimental data, technical rigor: Top 1%

Significance to chemistry researchers in this and related fields: Top 5%

Broad interest to other researchers: Top 5%

Novelty: Top 5%

Is this research study suitable for media coverage or a First Reactions (a News & Views piece in the journal)?: Yes

Reviewer: 2

Recommendation: Publish in ACS Central Science after minor revisions noted.

Comments:
In the manuscript by Wang et al., the authors investigate N-glycans on various IgG subtypes. The paper is well written, thorough, and the figures are great. I recommend publishing with the following minor edits:
- The synopsis should perhaps be expanded so as to be closer to the 60 word limit

Answer: Thank you for your suggestion. Based on your input, we have updated the synopsis as follows:
"This study introduces a nano-HILIC-LC-MS/MS method to analyze IgG glycosylation at the glycopeptide level, revealing unique structural glycosylation patterns across IgG subclasses. Distinct structural glycosylation signatures, particularly in galactose branching, are identified between subclasses, enhancing our understanding of IgG function and offering new insights into recombinant IgG for therapeutic use."

- It might be useful to include b/y ions in Figure 2

Answer: Thank you for your suggestion. We have incorporated several important b and y ions in Figure 2 of the revised manuscript. Additionally, detailed MS2 fragmentation data generated by Byonic are presented in the new Figure S1, as shown below. This includes HCDpdEThcD fragmentation with a comprehensive array of b/y and c/z ions for the glycopeptides depicted in Figure 2.

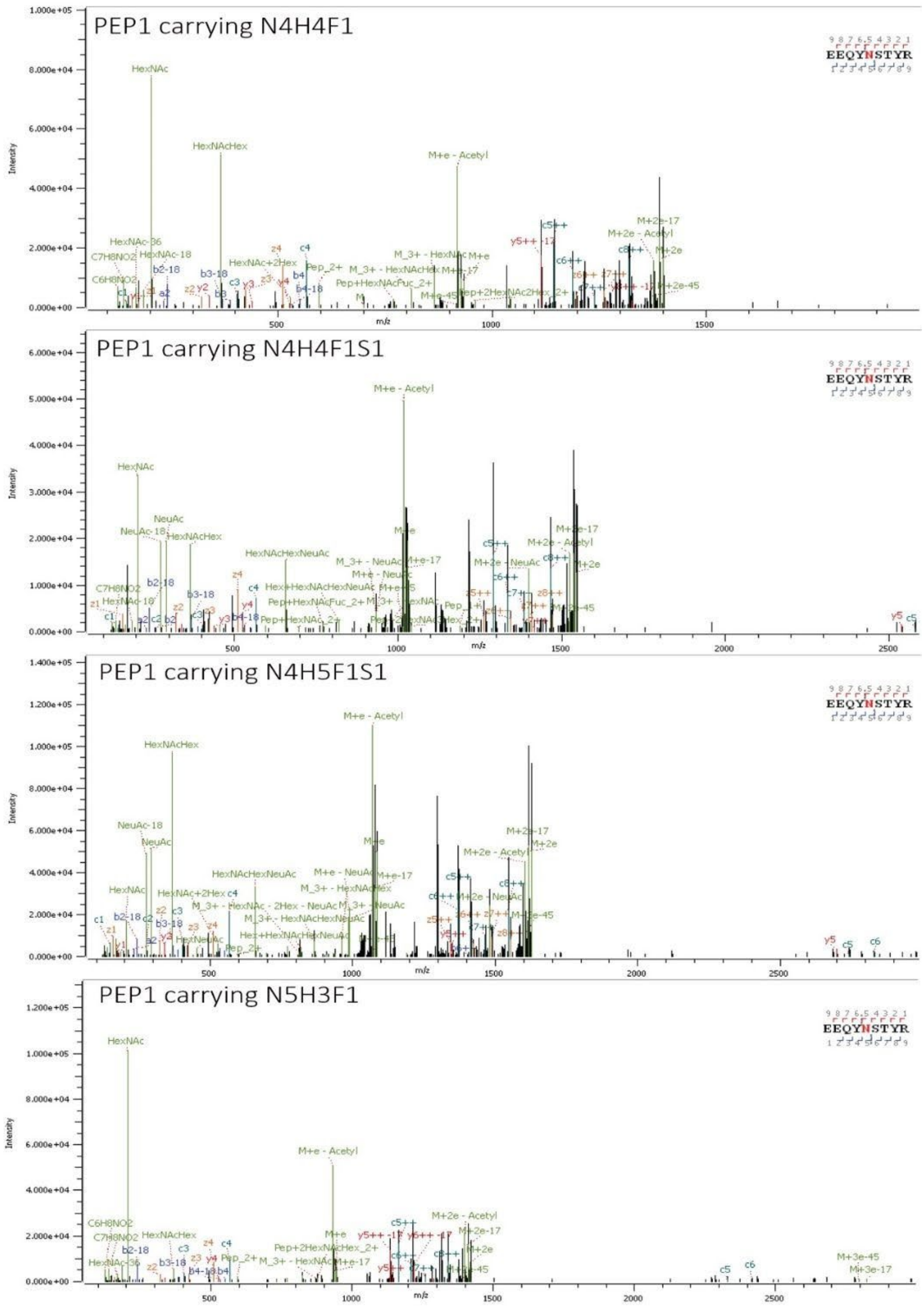

*"**Figure S1. HCDpdEThcD fragmentation of IgG1 glycopeptides.** Demonstrated here are PEP1 (sequence EEQYNSTYR) carrying glycans N4H4F1, N4H4F1S1, N4H5F1S1, and N5H3F1. As can be seen, a comprehensive range of b/y and c/z ions informs on the peptide identity."* - HILIC is mentioned throughout the text but is not included in the methods; please add

Answer: Thank you for your suggestion. Initially in the abstract, we have added the full name when HILIC is first mentioned. The content of the abstract has been modified as follows: *"Here, by development of a nano-hydrophilic interaction chromatography (HILIC)-LC-MS/MS method capable of unravelling glycan structure at the glycopeptide level…"*

Based on your suggestion, we have also provided a more detailed description of the HILICrelated sections in the Methods part of the main text, as shown below:

"HILIC column Preparation

The preparation of the HILIC column involved two steps: 1) the production of the frit-capillary, and 2) HILIC column packing. For step 1): the frit solution was prepared in a glass vial by mixing 300 µL potassium silicate (PQ Europe) with 100 µL formamide (Merck). After adding formamide, the solution was vortexed immediately. After that, one end of the capillary (75 µm ID, 40 cm) was dipped in the frit solution for a few seconds to provide a frit of ~1 cm. To check whether the frit was transported into the capillary, we used a microscope (Olympus) with a separate source of light (Schott, KL 1500). The capillaries were placed in a clean glass vessel with the frit end facing down and put in an oven for 1 h at 100 °C. During this step, the frit was polymerized. For step 2): capillaries with a frit on one end are washed twice with methanol. A slurry was then prepared by adding HILIC material (HALO penta-HILIC, 2.7 µm diameter, Advanced Materials Technology, USA). to ACN with 0.1% FA, which was subsequently packed into the capillary under a helium pressure of 100 bar with magnetic stirring. The capillary column was then consolidated at a liquid pressure of 800 bar (water or 80% methanol), trimmed to 25 cm, and set aside for use."

- Page 7, line 40, there's an additional "with" in the text

Answer: We appreciate the critical reading. However, going through all the instances of "with" within our text, we could not find the erroneous one.

- Ion intensity in the methods is listed as "5 x 103" and I believe it should be 10^3?

Answer: Thank you for your observation. We have made this change in our revised manuscript.

Additional Questions:
Quality of experimental data, technical rigor: Top 5%

Significance to chemistry researchers in this and related fields: High

Broad interest to other researchers: High

Novelty: Moderate

Is this research study suitable for media coverage or a First Reactions (a News & Views piece in the journal)?: No

oc-2024-01157n.R2

Name: Peer Review Information for "Human IgG subclasses differ in the structural elements of their <i>N</i>-glycosylation"

Second Round of Reviewer Comments

Reviewer: 2

Comments to the Author

The authors have addressed all of my comments.

Reviewer: 1

Comments to the Author

The authors have sufficiently addressed my comments, and this manuscript is now suitable for publication from my perspective.

Author's Response to Peer Review Comments:

Dear Editor,

Thank you for accepting our manuscript. In response to your requests, we have made the following modifications: The abstract has been reduced to 200 words and the synopsis has been refined to more precisely describe the Table of Contents (TOC). The TOC has also been resized appropriately and placed on the last page of the manuscript.

Thank you again for considering our work.

Yours sincerely, on behalf of all authors,

Dr. Weiwei Wang, email: w.wang1@uu.nl

Prof. dr. Albert J.R. Heck, email: a.j.r.heck@uu.nl

Dr. Karli R. Reiding, email: k.r.reiding@uu.nl
